# 2‐Oxoglutarate Analog‐Based Biomolecular Tools for Exploring Structure–Activity Relationships in Nonheme Iron Enzymes

**DOI:** 10.1002/cbic.202500177

**Published:** 2025-08-14

**Authors:** Peter Windsor, Sourav Chatterjee, Anoop Rama Damodaran, Ambika Bhagi‐Damodaran

**Affiliations:** ^1^ Department of Chemistry University of Minnesota Twin Cities Minneapolis 55455 USA

**Keywords:** 2‐oxoglutarates, analogs, nonheme irons, prolyl hydroxylase domain 2, structure–activity relationships

## Abstract

2‐oxoglutarate (2OG)‐dependent nonheme iron (NHFe) enzymes constitute a family of enzymes that use 2OG and oxygen to hydroxylate unactivated C(*sp* ^3^)–H bonds. These enzymes are biologically important and therapeutically relevant due to their role in key cellular processes. However, selective targeting remains challenging due to high structural conservation in their active sites. Herein, two classes of 2OG analogs are rationally designed and used as tools to investigate the active site of a 2OG‐dependent NHFe enzyme, prolyl hydroxylase domain 2 (PHD2). Using an activity assay in conjunction with steady‐state kinetics, a new class of aryl‐conjugated 2OG analogs is identified that exhibits 12‐fold varied inhibition and competes with 2OG for the PHD2 active site. Immunoblot studies suggest that these analogs are biologically active and can target PHD2 intracellularly. Furthermore, computational modeling studies reveal that the analogs bind to the active site in a “flipped” conformation relative to 2OG, and functional group placement is responsible for their different inhibition capabilities. Mutagenesis studies further validate this unique binding mode and suggest several interactions that are crucial for inhibition. Overall, these studies provide a toolkit of 2OG analogs to establish structure–activity relationships and identify interactions that can be useful for PHD2 inhibitor design.

## Graphical abstract

2‐oxoglutarate (2OG) analogs are rationally designed to elucidate structure–activity relationships (SARs) in 2OG‐dependent nonheme iron (NHFe) enzymes. These analogs are tested against prolyl hydroxylase domain 2 (PHD2). Activity assays reveal that certain analogs compete with 2OG for the PHD2 active site. Computational and mutagenesis studies reveal crucial binding residues, demonstrating the utility of these analogs to derive SARs.

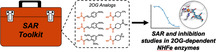

## 1 Introduction

2‐oxoglutarate (2OG)‐dependent nonheme iron (NHFe) enzymes are a diverse family of enzymes that are ubiquitously found in nature.^[^ ^1^ ^]^ These enzymes use an iron center along with several cosubstrates, like 2OG and oxygen (O_2_), to catalyze the hydroxylation of unactivated C(*sp* ^*3*^)–H bonds on amino acids and nucleic acids.^[^ ^2^, ^3^ ^]^ These hydroxylation reactions are paramount in regulating human biological processes such as DNA repair,^[^ ^4^, ^5^ ^]^ histone modification,^[^ ^6^, ^7^ ^]^ and hypoxia signaling.^[^ ^8^, ^9^ ^]^ Due to their role in these critical processes, dysfunction of these enzymes is suggested to lead to a broad range of pathologies including cancers and neurodegenerative disorders.^[^ ^10^, ^11^ ^]^ These diseases underscore the importance of 2OG‐dependent NHFe enzymes in maintaining cellular homeostasis and their potential as therapeutic targets.^[^ ^12^, ^13^ ^]^ The primary targeting approach for these enzymes focuses on developing small molecule inhibitors that compete with 2OG for the active site, but selectively targeting these enzymes remain challenging due to high structural conservation between 2OG binding sites.^[^ ^14^, ^15^, ^16^, ^17^ ^]^ Developing selective inhibitors can be a time intensive process, often requiring extensive optimization to achieve high target selectivity while minimizing off‐target effects. Off‐target effects can be particularly problematic as these enzymes and their byproducts are also involved in central metabolic processes such as structural protein synthesis and the tricarboxylic acid (TCA) cycle.^[^ ^16^, ^18^, ^19^ ^]^ These challenges highlight the need for chemical tools to streamline the development of these inhibitors.

2OG analogs have emerged as one set of tools that can be useful for establishing structure–activity relationships (SARs) between specific chemical motifs and active sites. These small molecules share structural similarity with 2OG but contain additional functional groups or substitutions that often render them catalytically inactive. However, they still compete with 2OG for the active site, thereby disrupting enzymatic activity.^[^ ^20^, ^21^, ^22^, ^23^, ^24^ ^]^ By systematically modifying or retaining certain functional groups, these analogs can be used to find motifs for selective inhibitor discovery and establish SARs.^[^ ^21^, ^25^ ^]^ Easy implementation of these analogs into biochemical assays make them effective tools for investigating the active site and inhibition of 2OG‐dependent NHFe enzymes.^[^ ^23^, ^25^ ^]^ For example, McDonough and coworkers have designed a 2OG analog inhibitor to be selective between two human hypoxia signaling enzymes, factor inhibiting hypoxia‐inducible factor (HIF) (FIH) and prolyl hydroxylase domain 2 (PHD2). The analog was highly selective for FIH by exploiting a unique nonpolar region in FIH's 2OG binding site, implying that this motif could be utilized in future inhibitor development.^[^ ^25^ ^]^ While a few 2OG analogs have been investigated for use in various enzymes, significant unexplored chemical space remains.

Prolyl hydroxylase domains (PHDs) are a class of NHFe enzymes implicated in human hypoxia signaling and have garnered significant interest as targets for inhibitor development due to the role of hypoxia signaling in various diseases.^[^ ^26^, ^27^, ^28^, ^29^, ^30^ ^]^ Creating molecular tools, such as novel 2OG analogs, could be beneficial to aid in the development of these inhibitors. Herein, we explore the use of a set of novel 2OG analogs to investigate the active site of human hypoxia signaling 2OG‐dependent NHFe enzyme, PHD2. We present the rational design and efficient syntheses for two distinct classes of novel 2OG analogs. Of these two classes, one class of unsaturated aryl‐based *α*‐keto‐acid analogs exhibits 12‐fold varied IC_50_ values via a competitive binding mechanism against 2OG. We further demonstrate that these analogs have cellular utility through immunoblot studies. We also validate the analogs’ binding mode using a combination of computational modeling and mutagenesis studies that suggest these analogs adopt a “flipped” conformation in the PHD2 active site relative to traditional 2OG binding. Furthermore, our mutagenesis studies suggest that two binding pocket residues, M299 and W258, are essential for binding these analogs. Overall, this study demonstrates the ability of 2OG analogs to establish SARs for targeting 2OG‐dependent NHFe enzymes.

## 2 Results and Discussion

### 2.1 Rational Design and Synthesis of 2OG Analogs

**Figure 1:**
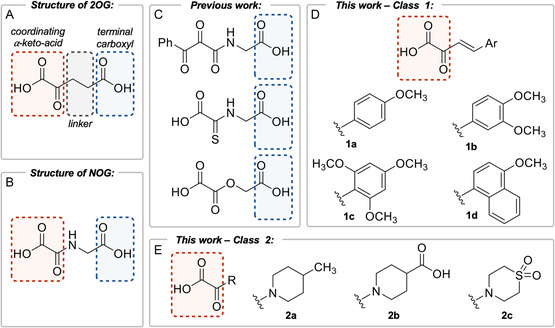
A) Structure of 2OG, the organic cofactor used by 2OG‐dependent NHFe enzymes, can be partitioned into distinct chemical moieties. The coordinating *α*‐keto‐acid (red box) binds to iron at the active site. The terminal carboxyl (blue box) forms a salt bridge with a positively charged residue in the binding pocket. The linker (gray box) links these two chemical moieties together. B) Structure of NOG, a well‐characterized, nonselective 2OG analog inhibitor of 2OG‐dependent NHFe enzymes. Contains a different linker group than 2OG making the analog catalytically inactive. C) Selected 2OG analogs from previously published work that maintain the terminal carboxyl group to form a salt bridge with a positively charged residue in the binding pocket. D) Highly conjugated, aryl‐based 2OG analogs that maintain the coordinating *α*‐keto‐acid group which are investigated in this work. E) Cyclic amine‐based 2OG analogs that maintain the coordinating *α*‐keto‐acid group which are investigated in this work.

Effective rational design of substrate analogs can be enhanced by a deeper understanding of the catalytic cycle and the substrate's interactions with the target enzyme. The first step of the catalytic cycle for 2OG‐dependent NHFe enzymes, like PHD2, is to bind 2OG to the iron center through the *α*‐keto‐acid moiety in a bidentate‐fashion. The enzyme then binds the substrate and O_2_ at the iron center, priming it for catalysis. O_2_ then performs a nucleophilic attack on the keto group of 2OG causing oxidative decarboxylation of 2OG into CO_2_ and succinate. This results in subsequent hydroxylation of the substrate and product release (Figure S1, Supporting Information).^[^ ^3^, ^31^, ^32^ ^]^ Based on binding mode and its role in catalysis, we note that the structure of 2OG can be partitioned into three distinct chemical moieties: 1) the *α*‐keto‐acid group which coordinates to the iron center in a bidentate‐fashion, 2) the terminal carboxyl group that forms a salt bridge with a positively‐charged residue in the active site (R383 in PHD2), and 3) a linker connecting the *α*‐keto‐acid and carboxyl group together (**Figure** **1**A). Inhibitor analogs investigated in previous studies have primarily focused on substitutions to the linker group and *α*‐keto‐acid group which make the analogs catalytically inactive.^[^ ^20^, ^21^, ^33^ ^]^ *N*‐oxalylglycine (NOG), a well‐characterized amide analog of 2OG, contains an amine substitution in the linker group. This substitution reduces the nucleophilicity of the keto group making the analog less capable of oxidative decarboxylation during catalysis (Figure 1B).^[^ ^34^ ^]^ Due to high‐structural similarity with 2OG, NOG functions as a pan inhibitor of 2OG‐dependent NHFe enzymes.^[^ ^33^, ^35^ ^]^ In addition to modifying the linker group, previous studies have also investigated 2OG analogs with modified *α*‐keto‐acid groups (Figure 1C). These analogs are substituted with motifs that still bind to iron in a bidentate‐fashion, however they do not undergo oxidative decarboxylation like 2OG, making them catalytically inactive.^[^ ^20^, ^21^ ^]^ While these studies have produced effective 2OG analogs there is still much chemical space left to explore, especially through further modification of the linker and terminal carboxyl group. Further exploring this chemical space can lead to the discovery of novel motifs that are useful for targeting 2OG‐dependent NHFe enzymes.

**Scheme 1:**
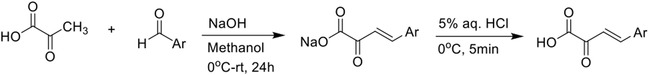
General synthesis of aryl‐based Class 1 analogs 1a–d.

**Scheme 2:**
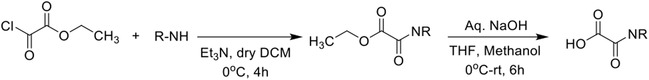
General synthesis of cyclic amine‐based Class 2 analogs 2a‐c.

In this study, we designed two classes of 2OG analogs featuring modified linker groups and lack terminal carboxyl groups while retaining the conventional *α*‐keto‐acid group for binding to the iron center (Figure 1D–E). Removing the terminal carboxyl group will prevent the possibility of salt bridge formation with positively‐charged residues in the active site, but alternative functionalization could potentially add interactions. Modifying the linker should make the analogs chemically inactive as in NOG. With these hypotheses, two classes of analogs were designed and synthesized. Class 1 analogs contained the *α*‐keto‐acid linked to various methoxy‐substituted aryl groups through a double bond linker (Figure 1D). We hypothesize this high degree of conjugation will reduce the nucleophilicity of the keto group making the analogs catalytically inactive. Additionally, the 2OG binding pocket of PHD2 is lined with hydrophobic residues and hydrogen bond donors suggesting the possibility for novel hydrophobic interactions or hydrogen bonds with the active site residues. These analogs were synthesized from pyruvic acid through an aldol condensation with various aryl methyl ketones. The compounds were then acidified to generate 1a‐d (**Scheme** **1**). Class 2 analogs contained the *α*‐keto‐acid linked to various cyclic amines. Similar to NOG, these analogs maintained an amine linker to be catalytically inactive, but contained a tertiary amine as opposed to the secondary amine of NOG. The terminal ends of these analogs were chemically diverse to probe a variety of interactions with the active site. Class 2 analogs were synthesized by coupling ethylchloroglyoxalate with various cyclic secondary amines followed by ester hydrolysis to generate 2a‐c (**Scheme** **2**). All of the synthesized analogs were obtained at decent yields (38%–56%) and their structures were confirmed by ^1^H NMR, ^13^C NMR, and high‐resolution mass spectrometry (Supporting Information).

### 2.2 Activity Screen and Inhibition Studies

**Figure 2:**
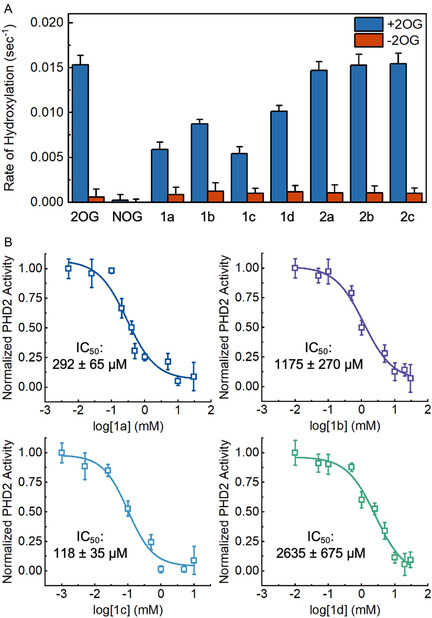
A) PHD2 activity screen monitoring inhibition by analogs (blue bars) or activation by analogs (red bars). Activity was monitored by hydroxylation assay. Error bars represent SD (n = 3). B) Dose‐response curves for the analogs and their respective IC_50_ values. Activity was monitored by hydroxylation assay. Error bars represent SD (n = 3).

We began our studies by screening both classes of analogs for inhibitory and catalytic properties using a PHD2 activity assay. This assay measures the conversion rate of a HIF‐1*α* peptide mimic—PHD2's biological substrate—into its hydroxylated form via matrix assisted laser desorption/ionization time‐of‐flight mass spectrometry. First, we monitored PHD2 activity in the presence of both 2OG and the analogs to determine if any of the analogs could inhibit PHD2 by competing with 2OG. NOG was used as a positive control for inhibition. These experiments were performed at cellularly relevant 2OG concentrations of 1 mM to saturate the PHD2 active site and mimic intracellular competition between 2OG and the analogs. The Class 1 analogs containing methoxy‐substituted aryl groups (1a‐d) inhibited PHD2 activity with varying efficacy (**Figure** **2**A, blue bars). Analogs 1a and 1c exhibited increased inhibition compared to analogs 1b and 1d. In contrast, cyclic amine‐based Class 2 analogs (2a‐c) did not inhibit PHD2 under the tested conditions. The observed differences in inhibition profiles of the two classes of analogs indicate that varying the terminal ends of 2OG analogs offers a viable strategy for tuning the activity of PHD2 inhibitors. The complete inhibition of PHD2 by NOG demonstrates that isosteric analogs with terminal carboxyl groups have increased potency compared to analogs with modified terminal ends.^[^ ^29^, ^33^, ^35^ ^]^ While the terminal carboxyl group promotes potency, it also increases the possibility of an analog being nonselective as the terminal carboxyl group interacts with a conserved basic residue in the active site of these enzymes.^[^ ^14^ ^]^

Previous studies have found that 2OG analogs containing *α*‐keto‐acid groups can activate hydroxylation catalysis in other 2OG‐dependent NHFe enzymes.^[^ ^23^, ^24^ ^]^ While we expected our modified linker groups to inactivate any potential catalysis, we moved forward with screening the analogs for hydroxylation activity. This screen was performed in the presence of the analogs but in the absence of 2OG so that hydroxylation activity could be attributed to the analogs. In the samples containing the analogs, minor amounts of hydroxylated peptide were detected. Interestingly, we also observed minor amounts of hydroxylated peptide in the negative control sample which lacked both 2OG and the analogs (Figure 2A, red bars). Previous work has shown that PHD2,^[^ ^36^ ^]^ along with other 2OG‐dependent NHFe enzymes,^[^ ^37^ ^]^ can be partially bound to 2OG upon purification, as 2OG is abundant during expression in *Escherichia coli* (500–1000 μM).^[^ ^38^ ^]^ The observed trace activity is likely catalyzed by residual 2OG that copurifies with PHD2 instead of catalyzed by the analogs. To verify this, we used a 2OG derivatization assay that allows for colorimetric detection of 2OG via 2,4‐dinitrophenylhydrazine (2,4‐DNPH) derivatization.^[^ ^39^ ^]^ We detected 27 μM 2OG in a 250 μM sample of purified PHD2 confirming that at least 10% of PHD2 copurifies with 2OG (Figure S2, Supporting Information). To further investigate if residual 2OG could activate hydroxylation catalysis, we monitored PHD2 activity by measuring succinate production in the absence of added 2OG, as succinate is a byproduct of 2OG decarboxylation during catalysis. Succinate was detected using a commercially available Succinate Glo assay.^[^ ^40^ ^]^ We observed that succinate levels increased over the course of the reaction, confirming that residual 2OG can activate hydroxylation catalysis and undergo oxidative decarboxylation (Figure S3, Supporting Information). Although the Class 2 analogs did not exhibit inhibition against PHD2 under the tested conditions, both classes of analogs were catalytically inactive, suggesting that these scaffolds could be further developed and tested as inhibitors against other 2OG‐dependent NHFe enzymes.

While our activity screen revealed that Class 1 analogs inhibited PHD2, we wanted to confirm that this inhibition occurred in a dose‐dependent manner, so the methoxy‐substituted aryl‐based analogs (1a‐d) were further evaluated for half‐maximal inhibitory concentration (IC_50_) at cellularly relevant 2OG concentrations of 1 mM (Figure 2B). Initial interpretation of the shape and slope of the dose‐response curves indicated a typical inhibition mechanism against PHD2.^[^ ^41^ ^]^ Additionally, the determined IC_50_ values agreed with the general trends observed in the activity screen with 1a and 1c exhibiting increased inhibition compared to 1b and 1d. Analogs 1b and 1d exhibited IC_50_ values in the low millimolar range (1175 ± 270 and 2635 ± 675 μM, respectively), whereas 1a and 1c exhibited IC_50_ values in the micromolar range (292 ± 65 μM and 118 ± 35 μM, respectively). The large differences in inhibition initially suggest that methoxy group positioning, along with steric bulk, can greatly influence inhibition of PHD2. The dose response of NOG was also evaluated under these conditions to serve as a reference inhibitor with a coordinating *α*‐keto‐acid and terminal carboxyl group. As expected, NOG exhibited high inhibition capacity with an IC_50_ of 4.3 ± 0.8 μM, demonstrating the impact of the salt bridge between the terminal carboxyl group and R383 in PHD2's binding pocket (Figure S4, Supporting Information). While this interaction clearly increases inhibition capacity, almost all 2OG‐dependent NHFe enzymes contain a positively‐charged residue in the 2OG binding site and targeting this interaction could lead to nonselective inhibition.

### 2.3 Steady‐State Kinetics and Immunoblot Studies

**Figure 3:**
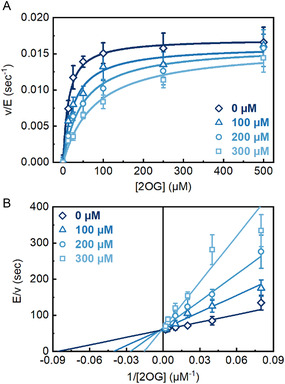
A) 2OG‐dependent steady‐state kinetics for inhibition of PHD2 at different concentrations of 1c (0, 100, 200, and 300 μM). Activity was monitored by hydroxylation assay. Error bars represent SD (n = 3). B) Lineweaver–Burke plots for inhibition of PHD2 at different concentrations of 1c (0, 100, 200, and 300 μM). Error bars represent SD (n = 3).

Next, we wanted to confirm that the analogs were in direct competition with 2OG. We performed 2OG‐dependent steady‐state kinetics to confirm the mechanism of inhibition and determine the inhibition constant (*K*_i_) of the two most potent 2OG analogs (1a and 1c). Michaelis–Menten plots and Lineweaver–Burk plots were used to confirm the mechanism of inhibition. For both analogs, the Michaelis–Menten plots (**Figure** **3**A and S5A, Supporting Information) showed that the 2OG Michaelis constant (*K*_M_(2OG)) increased with increasing inhibitor concentration, but *k*_cat_ changed minimally, indicating that these analogs were competitive against 2OG (Table S1 and S2, Supporting Information). Lineweaver–Burk plots further confirmed this observation as the trendlines intersected at the ordinate (Figure 3B and S5B, Supporting Information). The *K*_i_ values obtained from the plots for 1a and 1c were 140 ± 10 μM and 70 ± 7 μM, respectively, confirming that analog 1c is the more potent analog inhibitor as suggested by the IC_50_ values.

The combination of our dose‐response and steady‐state kinetics experiments suggested the analogs could compete with 2OG at cellularly relevant 2OG concentrations. Additionally, many cell‐permeable PHD2 inhibitors exhibit structural similarities to the analogs, such as being highly conjugated and having functionalized aryl groups.^[^ ^29^, ^42^ ^]^ We were therefore intrigued by the cellular utility of these analogs. In the cell, PHD2 catalyzes hydroxylation of HIF‐1*α*, which results in polyubiquitination and subsequent degradation of HIF‐1*α*. Inhibiting PHD2 prevents hydroxylation and causes HIF‐1*α* to accumulate, making HIF‐1*α* an appropriate marker for evaluating intracellular PHD2 inhibition.^[^ ^42^ ^]^ To investigate if these analogs could intracellularly target PHD2, we treated HEK‐293T cells with increasing doses of the two most potent 2OG analogs (1a and 1c) and monitored HIF‐1*α* levels using immunoblot. We also treated the cells with dimethyloxalylglycine (DMOG), a potent, cell‐permeable precursor of NOG that has been extensively used as a reference for intracellular PHD2 inhibition.^[^ ^43^, ^44^, ^45^ ^]^ Cells treated with DMOG started accumulating noticeable HIF‐1*α* levels at 500 μM and continued to increase up to the highest dose of 2000 μM (Figure S6A, Supporting Information). HIF‐1*α* accumulation was not detected in the cells treated with 1a (Figure S6B, Supporting Information), but the cells treated with 1c exhibited noticeable HIF‐1*α* accumulation at 2000 μM (Figure S6C, Supporting Information), suggesting that PHD2 was inhibited from hydroxylating HIF‐1*α* and that these analogs can compete with 2OG intracellularly. While the analogs were observed to be less effective than DMOG at causing HIF‐1*α* accumulation, our results imply that these analogs are cell‐permeable and have intracellular function. This experiment also further implicates 1c as being a more potent inhibitor relative to 1a.

### 2.4 Computational Modeling and Binding Mode Validation

**Figure 4:**
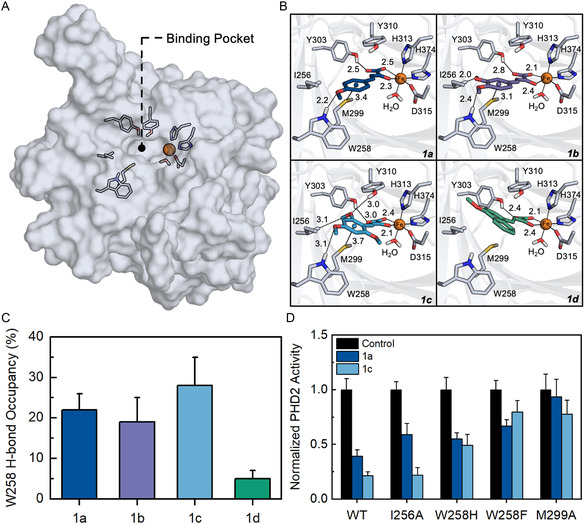
A) Surface representation of the PHD2 protein structure that was used for docking experiments (PDB: 2HBU). BIQ was removed from the structure to depict the binding pocket where the analogs were docked. The iron center is shown in orange and residues around the binding pocket are shown in gray. B) Binding modes of the analogs predicted from docking calculations. Analogs are shown in different colors. Dotted lines are contacts that the analogs make with residues in the binding pocket. Labeled distances are in angstroms (Å). C) Average hydrogen bond occupancy between W258 and 2OG analogs from MD trajectories. Error bars represent SD (n = 3). D) Activity of PHD2 mutants in the presence of analogs. Activity was monitored by hydroxylation assay. Error bars represent SD (n = 3).

The differences in inhibitory capacity demonstrated by our dose‐response and steady‐state kinetics experiments prompted us to further investigate the binding interactions between the analogs and PHD2 in an effort to establish a SAR. While we expected the *α*‐keto‐acid to bind to the iron center like 2OG, we were curious about which residues interacted with the modified terminal groups of the analogs and if these interactions were responsible for differential inhibition. To explore these questions, we turned to molecular docking to computationally predict the binding modes of the analogs. The docking studies were performed using the Glide module of the Maestro modeling suite. A crystal structure of PHD2 bound to BIQ (PDB: 2HBU), a known PHD2 inhibitor, was used as the docking receptor for these studies (**Figure** **4**A). BIQ and Class 1 analogs have structural similarities, such as being highly conjugated and having functionalized aryl groups, suggesting that this receptor model can predict accurate binding modes. The structure was validated by docking 2OG, NOG, and BIQ to the active site to ensure accurate predictions of known binding modes. These control molecules were modeled accurately and exhibited expected interactions with PHD2, such as formation of a salt bridge with R383 and hydrogen bonding with Y329, confirming the model was valid for docking (Figure S7, Supporting Information).

Upon docking the analogs, we observed that the *α*‐keto‐acid moiety was predicted to interact with the iron center as anticipated. Interestingly, the analogs adopted a “flipped” binding mode relative to 2OG and NOG with the modified terminal groups oriented toward the open side of the active site as opposed to buried in the protein core (Figure S8, Supporting Information). This suggests that the methoxy‐substituted aryl rings of the analogs are too bulky to bind in the typical 2OG orientation, forcing them to adopt an alternative binding mode. The solvent exposed side of PHD2's active site is still relatively inaccessible compared to other 2OG‐dependent NHFe enzymes, and as a result, the analogs make unique interactions with amino acids that are not observed in the binding modes of 2OG and NOG (Figure 4B). For example, Y303 is predicted to form a hydrogen bond with the carboxyl of the *α*‐keto‐acid in all of the analogs’ binding modes. Furthermore, M299 makes a contact with the aryl group of the analogs, suggesting the role of a potential methionine‐aromatic interaction in stabilizing analog binding. Methionine‐aromatic interactions are common motifs found in protein structures,^[^ ^46^, ^47^, ^48^ ^]^ and they have also been shown to play critical roles in promoting substrate and inhibitor binding.^[^ ^49^, ^50^ ^]^ The energies associated with these interactions are reported to be comparable to salt bridges.^[^ ^51^, ^52^, ^53^ ^]^ It is noteworthy that these analogs lack the conventional terminal carboxyl group of 2OG (Figure 1A), which typically interacts with positively charged residues in the binding pocket. However, the observed binding mode implies that a methionine‐aromatic interaction may be able compensate for the absence of this interaction.

The predicted binding modes also demonstrate that the positioning of the methoxy groups around the aryl ring allow for changes in hydrogen bonding and hydrophobic contacts which may contribute to the differential inhibition capability of these analogs. The *p*‐methoxy group forms a hydrogen bond with W258 in the binding modes of 1a, 1b, and 1c, but 1d possesses too much steric bulk forcing the methoxy group away from W258. The absence of this hydrogen bond, in addition to the steric bulk, is likely responsible for the weak inhibitory capacity of 1d. Alternatively, 1c has an *o*‐methoxy group which is predicted to hydrogen bond to Y310. No other analogs possess this functionalization, and as a result, do not exhibit this interaction. We believe that this hydrogen bond could be responsible for the higher potency of 1c compared to the other analogs. The terminal ends of the analogs also consistently make hydrophobic contacts with I256 implicating hydrophobic packing as being important for analog binding.

While our docking studies predicted reasonable binding modes for the analogs, we were further intrigued by the specific interactions promoting binding and differential inhibition. To gain structural insight, we performed molecular dynamics (MD) simulations of apo‐PHD2 and PHD2 bound to the analogs. Starting structures for the bound simulations were taken from docking calculations. All systems were independently simulated in triplicate for 200 ns to ensure adequate sampling of the system. Structural stability and equilibration of the systems were determined by monitoring the root mean square deviation (RMSD) and root mean square fluctuation (RMSF) of the C*_α_* atoms. The RMSD profiles revealed that the bound simulations reached equilibration within 25 ns, while the apo simulations exhibited large variation and did not reach a stable state on the 200 ns time scale (Figure S9, Supporting Information). Furthermore, the bound simulations exhibited a stable RMSD around 2.5 Å, whereas the apo simulations exhibited a variable range from 2‐5 Å. The observed stability of the bound simulations can be attributed to the interactions between the analogs and amino acids which prevent significant deviation of the C*_α_* atoms. The RMSF profiles further revealed that the analogs reduced fluctuations in three specific loop regions (L1–L3) of the protein which were adjacent to the binding pocket (Figure S10 and S11, Supporting Information). We observed that the analogs improved hydrophobic packing in the binding pocket resulting in reduced loop fluctuation.

We further analyzed the MD simulations to understand specific interactions that were important for analog binding such as hydrogen bonds and hydrophobic contacts. MD simulations confirmed our docking predictions and revealed that the *p*‐methoxy group of analogs 1a, 1b, and 1c formed a hydrogen bond with W258 for a significant part (≈20%) of the simulation, whereas the 1d only formed this hydrogen bond for 5 ± 2% of the simulation (Figure 4C). The steric bulk of 1d prevented this hydrogen bond from persisting, reinforcing the hypothesis that the weak inhibition by 1d can be attributed to steric bulk. Additionally, the hydrogen bond between the coordinating carboxyl of all of the analogs and Y303 was observed for a majority of the simulation (≈60%), suggesting that this hydrogen bond may be crucial for stabilizing analog binding. The docking results also predicted the *o*‐methoxy group of analog 1c to hydrogen bond with Y310, and this interaction was observed for 11 ± 3% of the simulation. This hydrogen bond may contribute to the stronger potency of 1c as the other analogs do not possess this functionalization. In addition to hydrogen bonding, we also observed hydrophobic contacts in the binding pocket. Our docking poses suggested the possibility of a methionine‐aromatic interaction persisting between M299 and the aryl group of the analogs. MD simulations revealed that the sulfur of M299 and the aryl groups were within the necessary distance (≤6.5 Å) for this interaction to occur for a large majority of the simulation, suggesting the possibility of this unique interaction in stabilizing analog binding (Figure S12, Supporting Information).

We were encouraged by our computational studies to experimentally validate the predicted binding modes and interactions of the analogs using mutagenesis studies. We constructed five mutants (I256A, W258F, W258H, M299A, and Y310T) and monitored their activity using our hydroxylation assay in the presence of 1a and 1c to verify the importance of specific amino acids contributing to analog binding and inhibition (Figure 4D). The Y310T mutant was constructed to probe the potential hydrogen bond between the *o*‐methoxy group of 1c and Y310. However, hydroxylation activity was not observed with this mutant, likely due to perturbing interactions with the substrate, making further evaluation not possible.^[^ ^54^ ^]^ The mutant was constructed to probe potential hydrophobic interactions with the methoxy‐substituted aryl rings. Inhibition by both analogs was mostly maintained, suggesting I256 does not play a large role in stabilizing binding. The W258F mutant was constructed to probe a potential hydrogen bond with the *p*‐methoxy group of both analogs, as the phenylalanine substitution should remove the hydrogen bond donating ability of the 258 residue. Inhibition by both analogs was significantly diminished, confirming that W258 stabilized binding. The W258H mutant was also constructed to probe the potential hydrogen bond with *p*‐methoxy group of both analogs, however, the histidine substitution should maintain the hydrogen bond donating ability of the 258 residue. While 1a and 1c demonstrated some inhibition against W258H PHD2 compared to W258F PHD2, we didn't observe the full extent of inhibition that was observed against WT PHD2. This indicated that the hydrogen bond between H258 and the analog was only partially present. To gain more structural insight about the hydrogen bonding behavior, we performed MD simulations of W258H PHD2 bound to 1a and 1c. We observed that H258 only hydrogen bonded to the analogs for ≈10% of the simulation as opposed to W258 which hydrogen bonded for ≈20% of the simulation. Visual analysis revealed that the reduction in hydrogen bonding was a result of increased conformational flexibility of H258 which led to unproductive hydrogen bonding confirmations. These unproductive confirmations typically arose from sidechain rotation along the C_β_—C_γ_ bond which oriented H258 away from the analog (Figure S13, Supporting Information). While histidine is capable of freely rotating along this bond, tryptophan's steric bulk and interactions with the surrounding residues prevented W258 from accessing these conformations. This observation was verified with sampling analysis of the *C*_α_—*C*_β_—*C*_γ_—*C*_δ_ dihedral bond (Figure S14, Supporting Information). Finally, the M299A mutant was constructed to probe a potential methionine‐aromatic interaction with the aryl ring of the analogs. We observed that the inhibitory properties of both analogs were decreased, confirming the importance of M299. Overall, our computational studies in combination with our mutagenesis studies reveal that W258 and M299 are essential residues for binding these analogs.

## 3 Conclusion

We have developed two classes of novel 2OG analogs to establish SAR for the active site of 2OG‐dependent NHFe enzymes. All these analogs are efficient and straightforward to synthesize and can be expanded for additional functional modification. As a proof of concept, these analogs are tested to probe the active site of PHD2, an enzyme crucial to human hypoxia signaling. Through a combination of biochemical activity assays, we identify Class 1 analogs containing aryl groups conjugated to *α*‐keto‐acid as competitive inhibitors of PHD2. Immunoblot studies further show that treatment of HEK‐293T cells with these inhibitors causes accumulation of HIF‐1*α*. Our computational and mutagenesis studies with these analogs reveal that hydrogen bonding with W258 and a unique methionine‐aromatic interaction with M299 are crucial for PHD2 inhibition. We suggest that targeting these interactions could be useful for designing PHD inhibitors. While our current study focused on PHD2, we believe that these analogs can be extended for use with other 2OG‐dependent NHFe enzymes.^[^ ^55^ ^]^ The *α*‐keto‐acid moiety present in these analogs ensures binding to the NHFe center, while the varied linker and terminal groups will provide crucial structure‐function information about the active site. Overall, this new class of 2OG analogs can be used to establish SARs and elucidate unique interactions for targeting 2OG‐dependent NHFe enzymes.

## Conflict of Interest

The authors declare no conflict of interest.

## Author Contributions

**Peter Windsor** performed all biochemical, computational, and cellular studies and analysis. **Sourav Chatterjee** performed all small molecule synthesis and characterization. **Anoop Rama Damodaran** and **Ambika Bhagi‐Damodaran** supervised the study. **Peter Windsor**, **Anoop Rama Damodaran**, and **Ambika Bhagi‐Damodaran** wrote the manuscript with inputs from all authors. **Peter Windsor** and **Sourav Chatterjee** contributed equally to this work.

## Supporting information

Supplementary Material

## Data Availability

The data that support the findings of this study are available from the corresponding author upon reasonable request.
