# Supplementary material for: 2‐Oxoglutarate Analog‐Based Biomolecular Tools for Exploring Structure–Activity Relationships in Nonheme Iron Enzymes
Source: Chembiochem. 2025 Aug 14;26(16):e202500177. doi: 10.1002/cbic.202500177 (PMC12432536; doi:10.1002/cbic.202500177)
Supplement: Supplementary file 1 — Supplementary Material [file CBIC-26-e202500177-s001.pdf]

Supporting Information

2-oxoglutarate analogue-based biomolecular tools for exploring structure-activity relationships in non-heme iron enzymes

Peter Windsor†, Sourav Chatterjee†, Anoop Rama Damodaran*, and Ambika Bhagi-Damodaran*

Department of Chemistry, University of Minnesota, Twin Cities, Minneapolis, MN-55455, USA.

*Corresponding author emails: rdanoop@umn.edu and ambikab@umn.edu

†These authors contributed equally

Table of Contents

Experimental Procedures……S2

Materials……S2

Site-directed mutagenesis……S2

Protein Expression and purification……S2

Inhibition screening, dose response, and steady-state kinetics assays……S3

Colorimetric Assay for 2OG Detection……S3

Succinate Glo™ Assay for Succinate Detection……S4

Cell culture, treatment, and lysis……S4

Immunoblotting……S4

Docking……S5

Molecular dynamics simulations……S5

Synthesis and characterization of 2OG analogues……S7

General information……S7

Experimental procedure for the synthesis of **1a-d**……S8

Experimental procedure for the synthesis of **2a-c**……S9

Analytical characterization of **1a-d** and **2a-c**……S11

^{1}H NMR, and ^{13}C NMR spectra of **1a-d** and **2a-c**……S13

Supplementary Figures……S20

Figure S1……S20

Figure S2……S21

Figure S3……S21

Figure S4……S22

Figure S5……S22

Figure S6……S23

Figure S7……S24

Figure S8……S24

Figure S9……S25

Figure S10……S25

Figure S11……S26

Figure S12……S27

Figure S13……S27

Figure S14……S28

Supplementary Tables……S29

Table S1……S29

Table S2……S29

Table S3……S29

Experimental Procedures

Materials

All chemicals were purchased from commercial vendors or synthesized as described. The sequence of the HIF-1α peptide mimic was DLDLEMLAPYIPMDDDFQL, and it was derived from native CODD of HIF-1α. The peptide (99% purity) was synthesized and purchased from Peptide Synthesis Services (University of Minnesota, Internal Service Organization).

Site-directed mutagenesis

Site-directed mutagenesis was used to create point mutations in PHD2 as previously described.[55] Briefly, WT-PHD2$_{181-426}$ was incorporated into pET-28a(+) expression vector with an N-terminal His$_6$ tag. Site-directed mutagenesis was performed on the WT PHD2 plasmid to create I256A, W258F, W258H, M299A, and Y310T mutations using Phusion site-directed mutagenesis kit (Thermofisher Scientific). Primer sequences can be found in Supporting Information (**Table S3**). A standard two-step protocol was used for PCR. Mutations were confirmed using classic Sanger sequencing at University of Minnesota Genomics Center (UMGC). T7 promoter (TAATACGACTCACTATAGGG) and T7 terminator (GCTAGTTATTGCTCAGCGG) were used for sequencing.

Protein expression and purification

WT-PHD2$_{181-426}$ and mutants were expressed and purified as previously described.[55,56] Briefly, BL21(DE3) E. coli (Thermofisher Scientific) were transformed with a pET-28a(+) expression vector. Cells were grown in 2XYT media supplemented with kanamycin (50 µg/mL) and grown to an OD$_{600}$ of 0.6-0.8. Protein expression was induced with 0.5 mM IPTG for 18 hours. Cells were harvested by centrifugation. Cells were resuspended and lysed via sonication in buffer (20 mM Tris HCl, 500 mM NaCl, and 5 mM imidazole at pH = 7.5) with Pierce Protease Inhibitor Tablets (Thermofisher Scientific). Cell lysate was purified using Ni-NTA affinity column (Cytiva) with running buffer (20 mM Tris HCl, 500 mM NaCl, and 5 mM imidazole at pH = 7.5) and elution buffer (20 mM Tris HCl, 100 mM NaCl, and 300 mM imidazole at pH = 7.5). Further purification was performed using a size-exclusion column (HiLoad Superdex 75pg 26/600, Cytiva). Protein purities (>95%) were assessed using SDS-PAGE and concentrations were determined using UV-Vis spectroscopy. Protein was exchanged into storage buffer (50 mM Tris HCl, 5% glycerol, pH = 7.5) and stored at 15 mg/mL at -80 °C.

Inhibition screening, dose response, and steady-state kinetics assays

PHD2 activity was assessed as previously described.[57] Briefly, the inhibition screening assay was carried out at 2 μM PHD2 (WT and mutants), 10 μM $(NH_4)_2Fe(SO_4)_2$, 100 μM HIF-1α peptide mimic, 2 mM sodium ascorbate, and 1 mM 2OG analogue. Activity was monitored in the presence of 2OG (1000 μM) and the absence of 2OG (0 μM). Dose response assays were carried out at 2 μM PHD2, 10 μM $(NH_4)_2Fe(SO_4)_2$, 100 μM HIF-1α peptide mimic, 1 mM 2OG, 2 mM sodium ascorbate, and varied 2OG analogue concentrations which were dependent on the analogues' potency. Steady-state kinetics assays were carried out at 2 μM PHD2, 10 μM $(NH_4)_2Fe(SO_4)_2$, 100 μM HIF-1α peptide mimic, varied 2OG concentrations (0 μM, 12.5 μM, 25 μM, 50 μM, 100 μM, 250 μM, and 500 μM), 2 mM sodium ascorbate, and varied 2OG analogue concentrations which were dependent on the analogues' potency. All reactions were performed in 50 mM Tris buffer (pH = 7.5) at 20 °C in a 1.7 mL reaction tube. MALDI-TOF mass spectrometry was used to monitor the conversion of the native peptide [(M + Na^+), 2276 m/z calculated, 2276 m/z observed] to the hydroxylated peptide [(M + O + Na^+), 2292 m/z calculated, 2292 m/z observed]. Reactions were quenched at different time points by spotting 1 μL of reaction mix directly on to the MALDI target plate with 1 μL of saturated 4-α-cyanohydroxycinnamic acid dissolved in 50% acetonitrile and 0.1% trifluoroacetic acid. Spots were analyzed using a positive reflectron method in a Daltonics Autoflex Speed MALDI-TOF mass spectrometer (Bruker). Percent conversion was calculated by comparing the relative intensities of the native and hydroxylated peptide species. All assays were performed in biological triplicate (n=3).

Colorimetric Assay for 2OG Detection

Residual 2OG in the PHD2 protein sample was detected using a colorimetric assay by derivatizing 2OG with 2,4-dinitrophenylhydrazine (2,4-DNPH) as previously described.[39] For the standard curve, solutions of 2OG (800 μM, 400 μM, 200 μM, 100 μM, 50 μM, 25 μM, 12.5 μM, and 6.25 μM) were prepared via serial dilution in 50 mM Tris buffer (pH = 7.5). To prepare the PHD2 sample, 250 μM PHD2 was incubated with 2.5 mM EDTA in 50 mM Tris buffer (pH = 7.5) overnight at 4 °C. PHD2 was separated out of solution using centrifugal filters (10 kDa cutoff followed by 3 kDa cutoff). The remaining flowthrough was then subject to derivatization with 2,4-DNPH. The standard 2OG solutions and flowthrough solution were diluted 2-fold with 4 mM-DNPH prepared in 2 M HCl and incubated at room temperature for 20 minutes. The solutions were then diluted 2-fold with 4 M NaOH and incubated at room temperature for 20 minutes. The solutions were then diluted 2-fold in 50 mM Tris buffer (pH = 7.5) for spectral acquisition. All spectra were recorded in an Cary8454 UV-Vis spectrophotometer (Agilent) at room temperature.

Succinate Glo™ Assay for Succinate Detection

Succinate in PHD2 reactions was detected using a commercially available Succinate Glo™ luminescence assay (Promega).[40] For the standard curve, solutions of succinate (15 μM, 7.5 μM, 3.75 μM, 1.88 μM, 0.94 μM, 0.47 μM, and 0.23 μM) were prepared via serial dilution in 50 mM Tris buffer (pH = 7.5). PHD2 reactions were carried out at 2 μM PHD2, 10 μM $(NH_4)_2Fe(SO_4)_2$, 100 μM HIF-1α peptide mimic, and 2 mM sodium ascorbate. The negative control reaction was absent of PHD2, and the positive control reaction contained 10 μM 2OG. All reactions were performed in 50 mM Tris buffer (pH = 7.5) at 20 °C in a 1.7 mL reaction tube. The reactions were quenched at different time points by transferring aliquots of the reaction to a white 384-well plate (Greiner) and adding an equal volume of Succinate Glo Reagent 1. The plate was mixed for 30 seconds and incubated at room temperature for 60 minutes. The solution was then diluted 2-fold with Succinate Glo Reagent 2 and incubated at room temperature for 20 minutes. The samples were monitored for luminescence using a Spark Microplate Reader (Tecan).

Cell culture, treatment, and lysis

HEK-293T cells were cultured in Dulbecco's Modified Eagle's medium (DMEM) (11965092, Gibco) supplemented with 10% Fetal Bovine Serum (F0926, Sigma) and 1% penicillin-streptomycin (Corning). The cells were maintained in a 37 °C incubator supplied with 5% CO_2. For treatment with 2OG analogues, cells were seeded 24 hours before transfection at a density of 2×10^5 cells/ml and then treated with the analogues at varying concentrations. Cells were incubated for 18 hours and then harvested by lysing in 1X cell lysis buffer (Cell Signaling Technology) and 1 mM phenylmethylsulfonyl fluoride (PMSF).

Immunoblotting

Proteins in cell lysate were quantified using the Pierce BCA Protein Assay Kit (Thermofisher Scientific), and concentrations were normalized to 2000 μg/mL. Proteins were separated in a Bolt 4-12% Bis-Tris Plus WedgeWell Gel (Thermofisher Scientific) and transferred onto a nitrocellulose membrane. The membrane was dried overnight at room temperature to fix the proteins to the membrane. The membrane was rehydrated in TBS and blocked using 5% BSA in TBST (TBS + 0.1% Tween20) for 1 hour at room temperature. The membrane was then incubated with primary antibodies (HIF-1α (D1S7W) and α-Tubulin (DM1A), Cell Signaling Technology) for 1 hour at room temperature. The membrane was washed 4 times with TBST and then incubated with secondary antibodies (IRDye 680RD (926-68070) and IRDye 800CW (926-32211), LI-COR) for 1 hour at room temperature. The membrane was washed 4 times with TBST and then dried at

37 °C for 1 hour. The membrane was imaged on an Odyssey M Imager (LI-COR) with excitation channels 700 nm (α-Tubulin) and 800 nm (HIF-1α). The images were processed and analyzed using Empiria Studio 3.0 (LI-COR).

Docking

Binding modes of the 2OG analogues were assessed using the Glide module of the Maestro modeling suite (Schrödinger).[58] A PHD2 crystal structure (PDB: 2HBU) obtained from the Protein Data Bank (PDB) was used to assess the binding modes of the 2OG analogues. The protein structure was prepared using the Protein Preparation module of Maestro. Protonation states of amino acids were determined at a pH of 7.4. Hydrogen bonds were optimized, and the structures were energy minimized using OPLS4 forcefield. All crystallized waters at a distance greater than 5 Å were deleted from the structures. The receptor grid was generated around the bound ligand (BIQ) in the crystal structure. The 2OG analogue structures were prepared and minimized in Avogadro using the GAFF forcefield. The analogues (**1a**, **1b**, **1c**, and **1d**) were docked using the default settings and the Standard Precision (SP) docking method.

Molecular dynamics simulations

Molecular dynamics simulations were performed as previously described.[59] Briefly, PHD2 crystal structure (PDB: 2HBU) complexed with the respective docking mode for each analogue were used as the starting structure for the simulations. All ligand distances were restrained to within 0.1 Å of their crystallographic distances (or distances from the docking mode) using a force constant of 100.0 kcal mol^{-1} $Å^{-1}$ to maintain the primary coordination sphere about iron. The system was simulated with the Amber ff14SB forcefield using the GPU-accelerated pmemd code of Amber16. The system was minimized using a seven-step process by systematically lowering restraints over the course of each step. Next, the system was heated linearly from 10.0 K to 298.0 K over 2.0 ns with all solute atoms subject to a restraint of 10.0 kcal/mol/$Å^2$. Equilibration of the system was carried out over 3.5 ns at a constant temperature of 298.0 K. All solute heavy atoms were subjected to restraints of 10.0 kcal/mol/$Å^2$ for the first 0.5 ns, and restraints were decreased geometrically every 0.5 ns until fully unrestrained. After equilibration of the system, unrestrained MD was performed at a constant pressure of 1 atm and constant temperature of 298.0 K. The restraints between the analogue and iron center were removed for the unrestrained portion of the simulation, but the restraints were maintained for the other ligands around the iron center. All systems were simulated in triplicate (n=3) for 200 ns. MD analysis (RMSD, RMSF, distances, and h-bonding) was performed using the cpptraj module of Amber16. The resulting values for each

simulation were averaged and reported with standard deviations. The visualization of the completed trajectories was carried out using PyMOL.

Synthesis and characterization of 2OG analogues

General Information

All reactions were carried out in the presence of air or in Standard Schlenk techniques under predried nitrogen. All reagents were purchased from commercial suppliers and used without further purification. ^{1}H NMR spectra were recorded on a Bruker Avance III HD 400 MHz NMR instrument at ambient temperature either in $CDCl_3$, CD_3OD, D_2O or DMSO-d6. ^{13}C NMR spectra were recorded at 100 or 125 MHz at ambient temperature. The chemical shifts were recorded in parts per million (ppm) with TMS as internal reference. ^{1}H NMR is reported as follows: chemical shift, multiplicity (s = singlet, d = doublet, dd = doublet of doublet, brs = broad singlet, t = triplet, q = quartet, m = multiplet), coupling constant and integration. Coupling constant (*J*) values are given in hertz (Hz). All ^{13}C NMR spectra were recorded with complete proton decoupling. Mass spectral data correspond to ESI-MS and are given in *m/z* unit. ESI HRMS were done on a Sciex X500R Mass Spectrometer. Analytical thin-layer chromatography (TLC) was carried out on Merck 20 × 20 cm silica gel 60-F_{254} plates. Column chromatography was performed with Teledyne Combiflash, silica gel 230-400 cartridge.

Experimental procedure for the synthesis of 1a-d

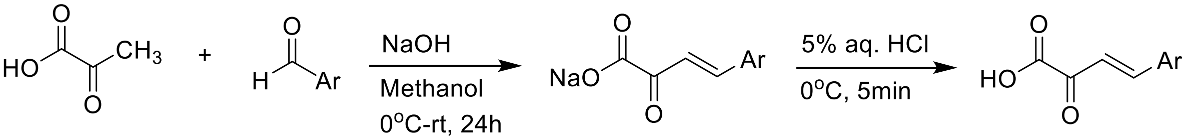

1a-d

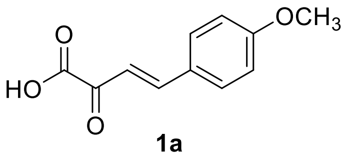

1a

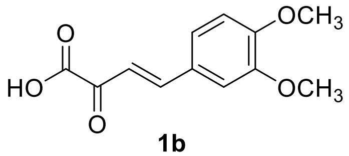

1b

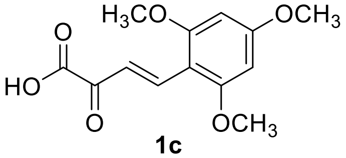

1c

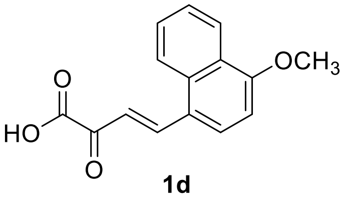

1d

Procedure for 1a-d

To a stirred solution of pyruvic acid (7.34mmol, 520µL) in methanol (2mL), a solution of NaOH (11mmol, 440mg) in methanol (3mL) was added to it dropwise over a period of 10min at 0°C. It was further stirred at 0°C for 60min and a precooled solution of *p*-methoxy benzaldehyde (7.34mmol, 1g) in methanol (1mL) was further added to it dropwise under cold condition. The reaction mixture was further stirred for 24h at room temperature. Precipitate filtered, washed with cold methanol (3 × 10mL) and then with diethyl ether (2 × 10mL). The solid was dried to afford sodium salt of corresponding α-keto-acid (660mg, 39%) as bright-yellow solid. The salt was further acidified with 5% aqueous HCl, precipitate filtered, washed with cold water and dried to isolate (*E*)-4-(4-methoxyphenyl)-2-oxobut-3-enoic acid (**1a**) in quantitative yield. Compounds (**1b-d**) were prepared following the same experimental protocol.

Experimental procedure for the synthesis of 2a-c

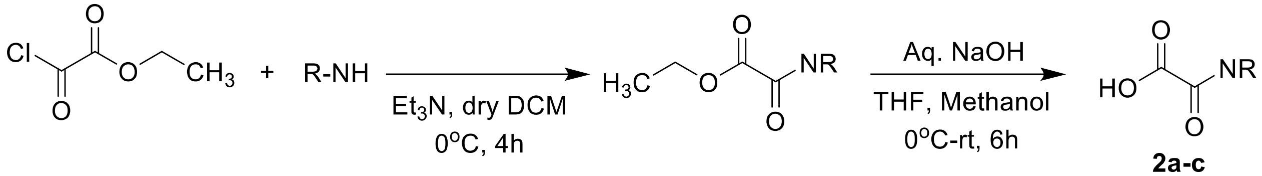

2a-c

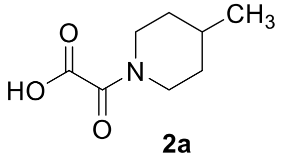

2a

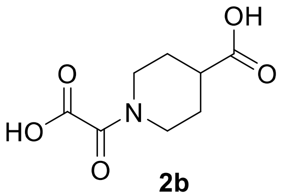

2b

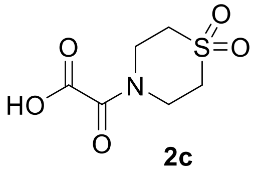

2c

Procedure for 2a

In a dry Schlenk tube, ethyl chloroglyoxylate (3.66mmol, 409μL) was added dropwise to a stirred solution of 4-methylpiperidine (4.03mmol, 476μL) in presence of triethyl amine (5.49mmol, 766μL) in anhydrous DCM (3.5mL) at 0 °C under nitrogen atmosphere. The reaction mixture was then stirred at 0 °C for 4h. Added cold water (20mL) and extracted with DCM (2 × 20mL). The corresponding amide ester was flash chromatographed on silica gel (230-400) eluting with 15-20% ethyl acetate in hexane and isolated as pale-yellow liquid (481 mg, 66%).

The amide-ester (2.0mmol, 400mg) was further hydrolyzed using aqueous NaOH (4.0mmol, 160mg in 3.0mL water) in THF-methanol (1:1, 7mL) under cold condition for 6h. THF-methanol mixture was dried, added water (10mL) and extracted with ethyl acetate (2 × 10mL). The aqueous layer was acidified using 5% aqueous HCl under cold conditions and extracted with 2% methanol in DCM (2 × 20mL). The dry sample was triturated with diethyl ether, filtered, and further washed with diethyl ether to provide pure 2-(4-methylpiperidin-1-yl)-2-oxoacetic acid (312 mg, 91%) as white solid.

Procedure for 2b

In a dry Schlenk tube, ethyl chloroglyoxylate (3.66mmol, 409μL) was added dropwise to a stirred solution of ethyl-4-piperidinecarboxylate (4.03mmol, 621μL) in presence of triethyl amine (5.49mmol, 766μL) in anhydrous DCM (3.5mL) at 0 °C under nitrogen atmosphere. The reaction mixture was then stirred at 0 °C for 4h. Added cold water (20mL) and extracted with DCM (2 × 20mL). The corresponding diethyl ester was flash chromatographed on silica gel (230-400) eluting with 20-25% ethyl acetate in hexane and isolated as clear liquid (593 mg, 63%).

The diethyl ester (1.94mmol, 500mg) was further hydrolyzed using aqueous NaOH (5.82mmol, 233mg in 3.0mL water) in THF-methanol (1:1, 7mL) under cold condition for 6h. THF-methanol mixture was dried, added water (10mL) and extracted with ethyl acetate (2 × 10mL). The aqueous layer was acidified using 5% aqueous HCl under cold conditions (pH ~ 5-6) and lyophilized. The dry mass was washed with 30% methanol in DCM and filtered through a sintered funnel. The filtrate was dried under reduced pressure, the dry sample was triturated and washed with diethyl ether, filtered, and dried to provide 1-(carboxycarbonyl)piperidine-4-carboxylic acid (168 mg, 43%) as white solid.

Procedure for 2c

In a dry Schlenk tube, ethyl chloroglyoxylate (3.66mmol, 409µL) was added dropwise to a stirred solution of thiomorpholine 1,1-dioxide (4.03mmol, 545mg) in presence of triethyl amine (5.49mmol, 766µL) in anhydrous DCM (3.5mL) at 0 °C under nitrogen atmosphere. The reaction mixture was then stirred at 0 °C for 4h. Added cold water (20mL) and extracted with DCM (2 × 20mL). The corresponding amide ester was flash chromatographed on silica gel (230-400) eluting with 50-70% ethyl acetate in hexane and isolated as white crystalline solid (508 mg, 59%).

The amide-ester (2.0mmol, 470mg) was further hydrolyzed using aqueous NaOH (4.0mmol, 160mg in 3.0mL water) in THF-methanol (1:1, 7mL) under cold condition for 6h. THF-methanol mixture was dried, added water (10mL) and extracted with ethyl acetate (2 × 10mL). The aqueous layer was acidified using 5% aqueous HCl under cold conditions (pH ~ 5-6). The resulted white precipitate was centrifuged, collected and dried. The dry sample was triturated and washed with diethyl ether, filtered, and dried to provide 1-(carboxycarbonyl)piperidine-4-carboxylic acid (205 mg, 46%) as white solid.

Characterization of 1a-d and 2a-c

(*E*)-4-(4-methoxyphenyl)-2-oxobut-3-enoic acid (1a)

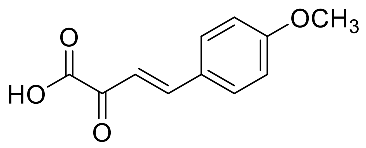

^{1}H NMR (400 MHz, CD_3OD) δ 7.84 (d, *J* = 15.2 Hz, 1H), 7.63 (d, *J* = 6.8 Hz, 2H), 7.19 (d, *J* = 15.2 Hz, 1H), 6.97 (d, *J* = 7.2 Hz, 2H), 3.83 (s, 3H). ^{13}C NMR (100 MHz, CD_3OD) δ 186.8, 164.3, 150.3, 132.2, 128.4, 120.0, 115.7 (2C), 56.0. HRMS(ESI) *m/z* calculated for $C_{11}H_{10}O_4$ $[M+Na]^+$: 229.0471, found: 229.0474, Error: 1.09 ppm.

(*E*)-4-(3,4-dimethoxyphenyl)-2-oxobut-3-enoic acid (1b)

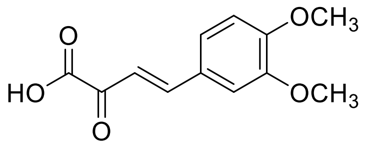

^{1}H NMR (400 MHz, CD_3OD) δ 7.81 (d, *J* = 16.1 Hz, 1H), 7.31 (d, *J* = 8.5 Hz, 2H), 7.25 (d, *J* = 16.1 Hz, 1H), 7.03 (d, *J* = 8.1 Hz, 1H), 3.89 (s, 6H). ^{13}C NMR (125 MHz, DMSO-d_6) δ 186.3, 165.0, 152.0, 149.1, 148.2, 126.6, 124.4, 119.3, 111.6, 110.8, 55.6 (2C). HRMS(ESI) *m/z* calculated for $C_{12}H_{12}O_5$ $[M+2Na]^+$: 281.0402, found: 281.0404, Error: 0.71 ppm.

(*E*)-2-oxo-4-(2,4,6-trimethoxyphenyl)but-3-enoic acid (1c)

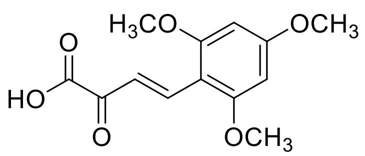

^{1}H NMR (400 MHz, $CDCl_3$) δ 8.60 (d, *J* = 16.0 Hz, 1H), 7.89 (d, *J* = 16.0 Hz, 1H), 6.11 (s, 2H), 3.93 (s, 6H), 3.88 (s, 3H). ^{13}C NMR (125 MHz, DMSO-d_6) δ 187.4, 165.6, 164.4, 161.8 (2C), 138.8, 119.9, 104.6, 91.1 (2C), 56.2 (2C), 55.7. HRMS(ESI) *m/z* calculated for $C_{13}H_{15}O_6$ $[M+Na]^+$: 289.0683, found: 289.0684, Error: 0.41 ppm.

(*E*)-4-(4-methoxynaphthalen-1-yl)-2-oxobut-3-enoic acid (1d)

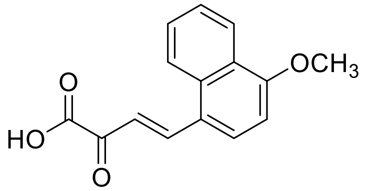

^{1}H NMR (400 MHz, CD_3OD) δ 8.71 (d, *J* = 15.8 Hz, 1H), 8.32 (d, *J* = 8.3 Hz, 1H), 8.23 (d, *J* = 8.6 Hz, 1H), 8.08 (d, *J* = 8.3 Hz, 1H), 7.68 – 7.64 (m, 1H), 7.57 – 7.54 (m, 1H), 7.41 (d, *J* = 15.8 Hz, 1H), 7.05 (d, *J* = 8.3 Hz, 1H), 4.09 (s, 3H). ^{13}C NMR (125 MHz, DMSO-d_6) δ 185.7, 164.8, 158.0, 143.2, 132.2, 128.1, 127.9, 125.9, 124.8, 122.6, 122.5, 122.3, 120.9, 104.9, 56.1. HRMS(ESI) *m/z* calculated for $C_{15}H_{13}O_4$ $[M+H]^+$: 257.0808, found: 257.8012, Error: 1.56 ppm.

2-(4-methylpiperidin-1-yl)-2-oxoacetic acid (2a)

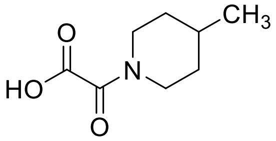

^{1}H NMR (400 MHz, D_2O) δ 4.22 – 4.16 (m, 1H), 3.68 – 3.63 (m, 1H), 3.21 – 3.14 (m, 1H), 2.80 (td, *J* = 12.9, 2.9 Hz, 1H), 1.76 – 1.66 (m, 3H), 1.24 – 1.05 (m, 2H), 0.93 (d, *J* = 6.4 Hz, 3H). ^{13}C NMR (100 MHz, D_2O) δ 168.7, 165.2, 47.0, 41.4, 33.7, 32.8, 30.0, 20.6. HRMS(ESI) *m/z* calculated for $C_8H_{13}NO_3$ $[M-H]^-$: 170.0822, found: 170.0804, Error: 10.88 ppm.

1-(carboxycarbonyl)piperidine-4-carboxylic acid (2b)

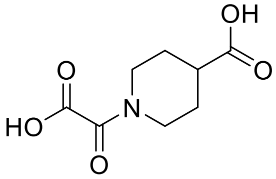

^{1}H NMR (400 MHz, D_2O) δ 4.19 – 4.16 (m, 1H), 3.73 – 3.69 (m, 1H), 3.30 – 3.23 (m, 1H), 3.00 – 2.93 (m, 1H), 2.79 – 2.72 (m, 1H), 2.06 – 2.02 (m, 2H), 1.75 – 1.59 (m, 2H). ^{13}C NMR (100 MHz, $CDCl_3$) δ 178.8, 169.7, 167.1, 45.7, 40.2, 40.0, 27.8, 26.9. HRMS(ESI) *m/z* calculated for $C_8H_{11}NO_5$ $[M-H]^-$: 200.0564, found: 200.0548, Error: 8.14 ppm.

1-(carboxycarbonyl)piperidine-4-carboxylic acid (2c)

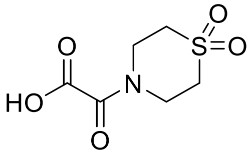

^{1}H NMR (400 MHz, D_2O) δ 4.07 – 4.04 (m, 1H), 3.97 – 3.94 (m, 1H), 3.86 – 3.83 (m, 2H), 3.67 – 3.64 (m, 2H), 3.38 (m, 2H). ^{13}C NMR (100 MHz, $CDCl_3$) δ 168.0, 167.1, 51.1, 50.6, 44.4, 39.0. HRMS(ESI) *m/z* calculated for $C_6H_{10}NO_5S$ $[M-H]^-$: 206.0128, found: 206.0108, Error: 10.00 ppm.

^{1}H and ^{13}C NMR spectra of 1a-d and 2a-c

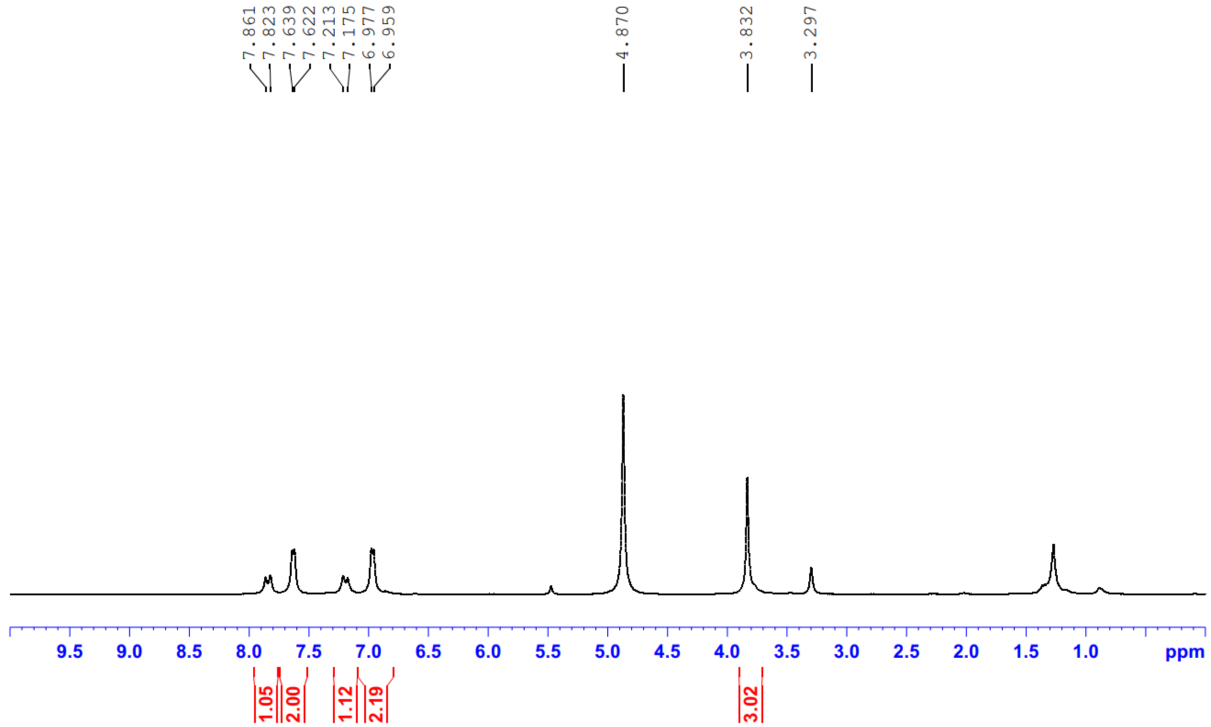

^{1}H NMR spectra of **1a** in CD_3OD

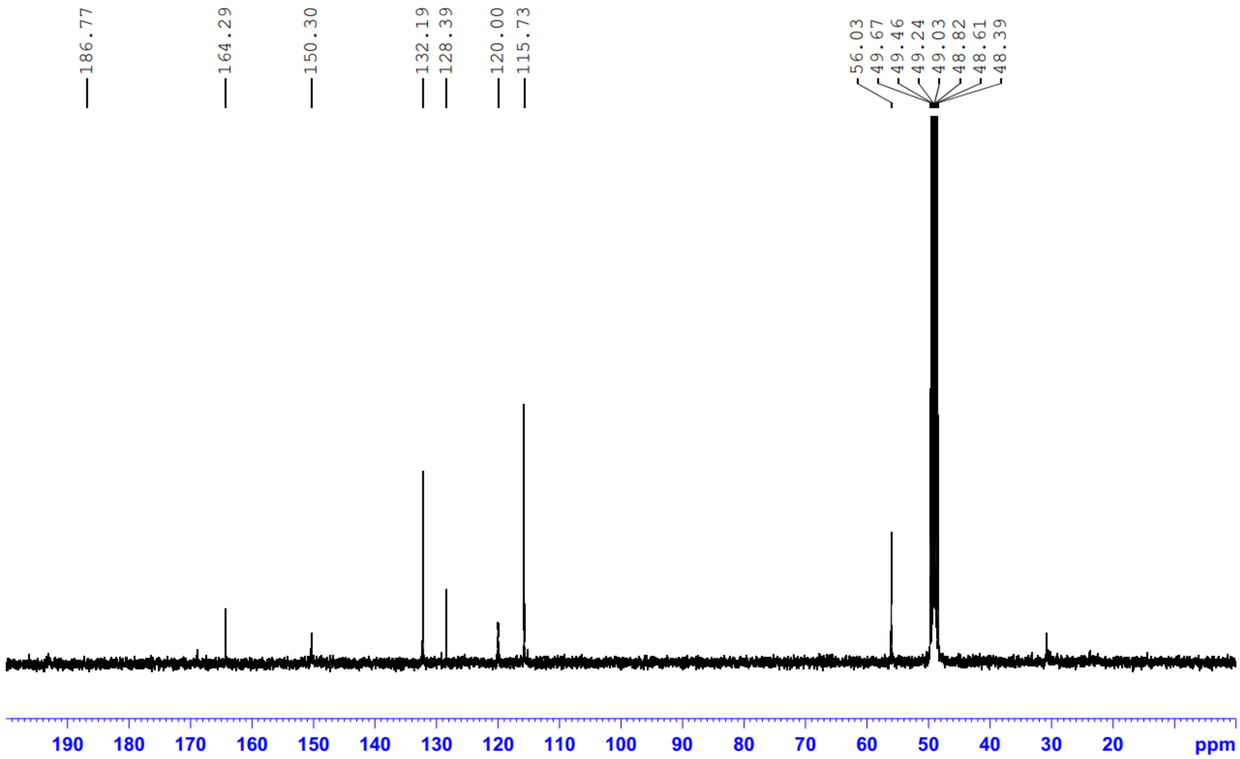

^{13}C NMR spectra of **1a** in CD_3OD

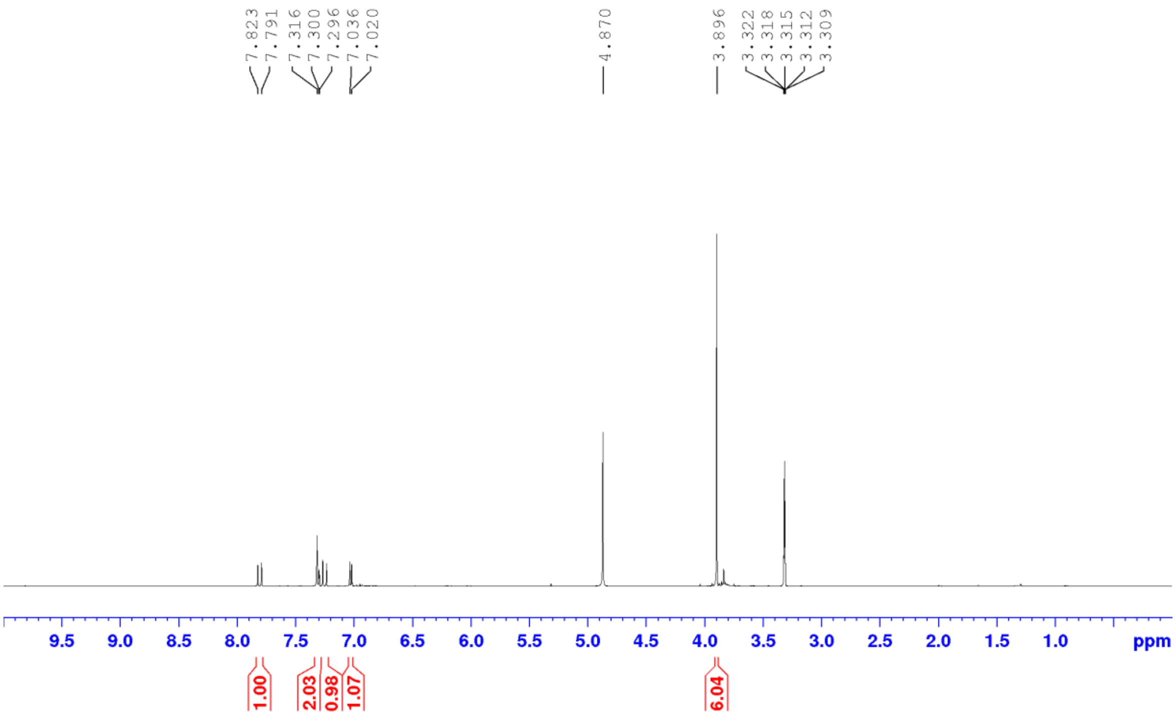

^{1}H NMR spectra of **1b** in CD_3OD

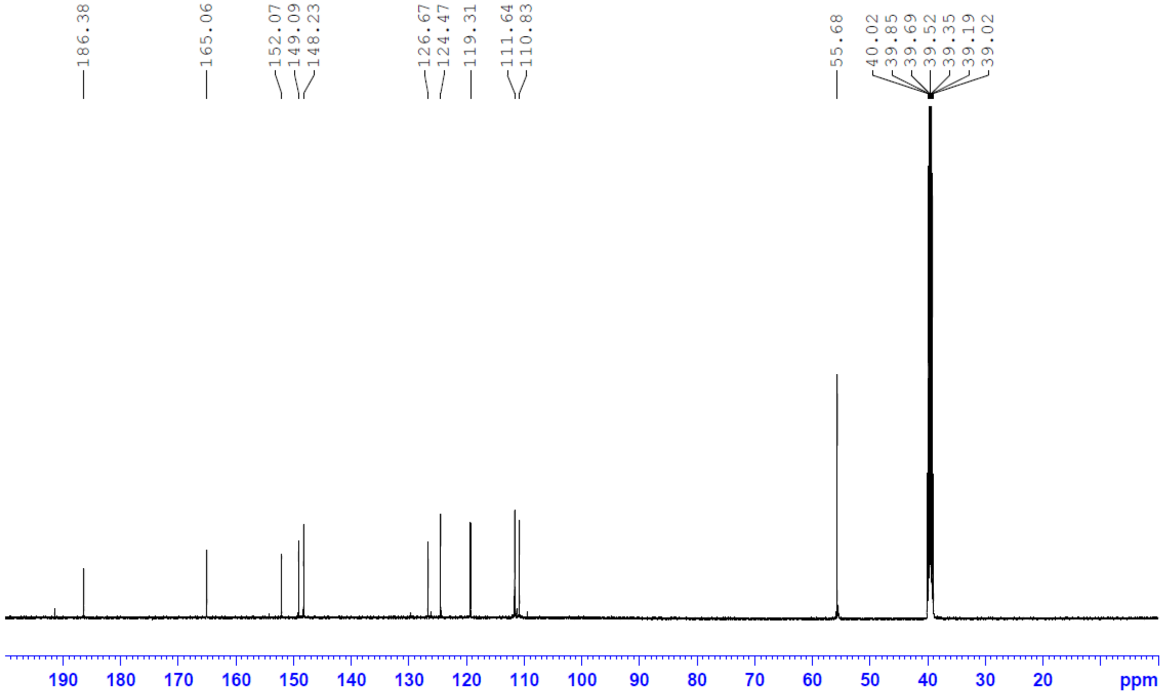

^{13}C NMR spectra of **1b** in DMSO-d6

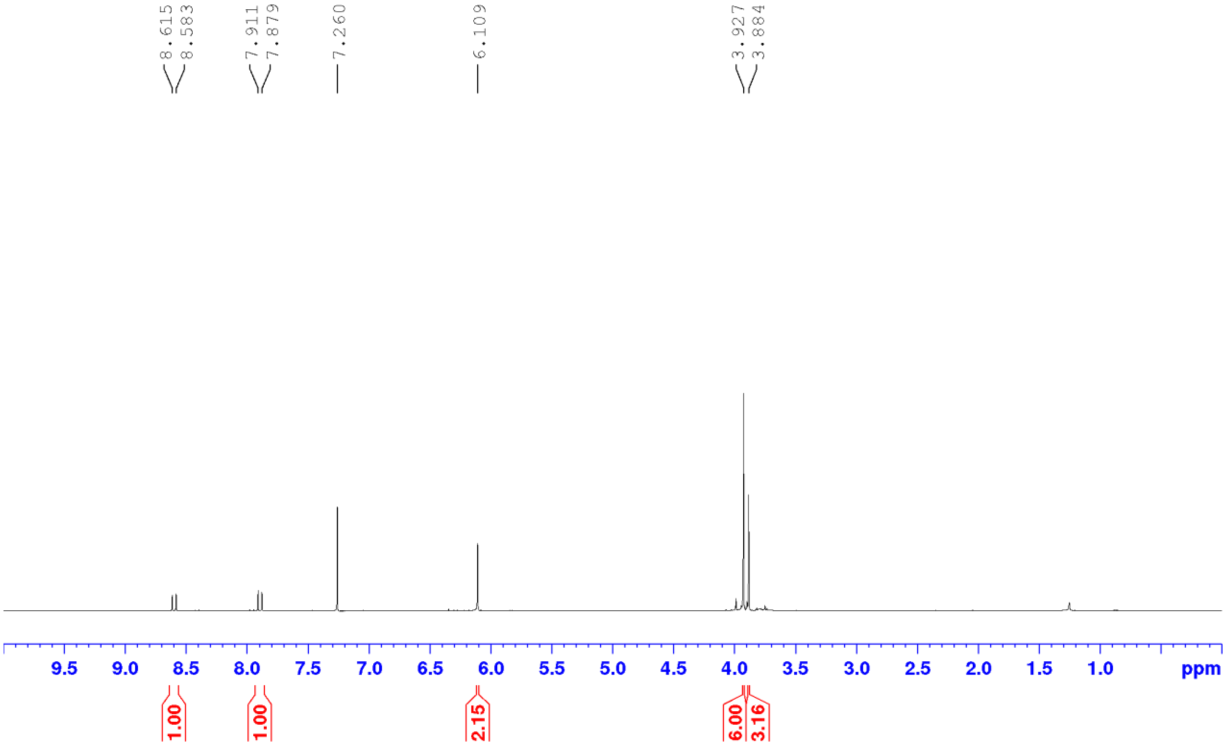

^{1}H NMR spectra of **1c** in $CDCl_3$

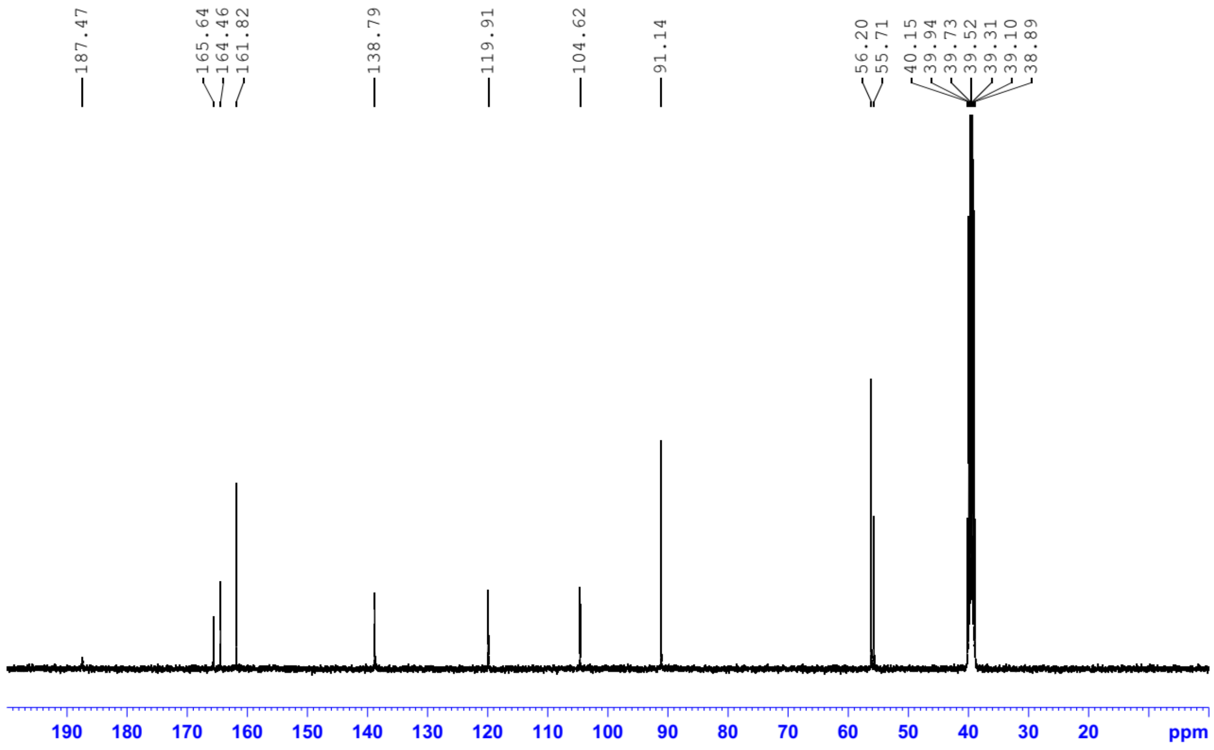

^{13}C NMR spectra of **1c** in DMSO-d6

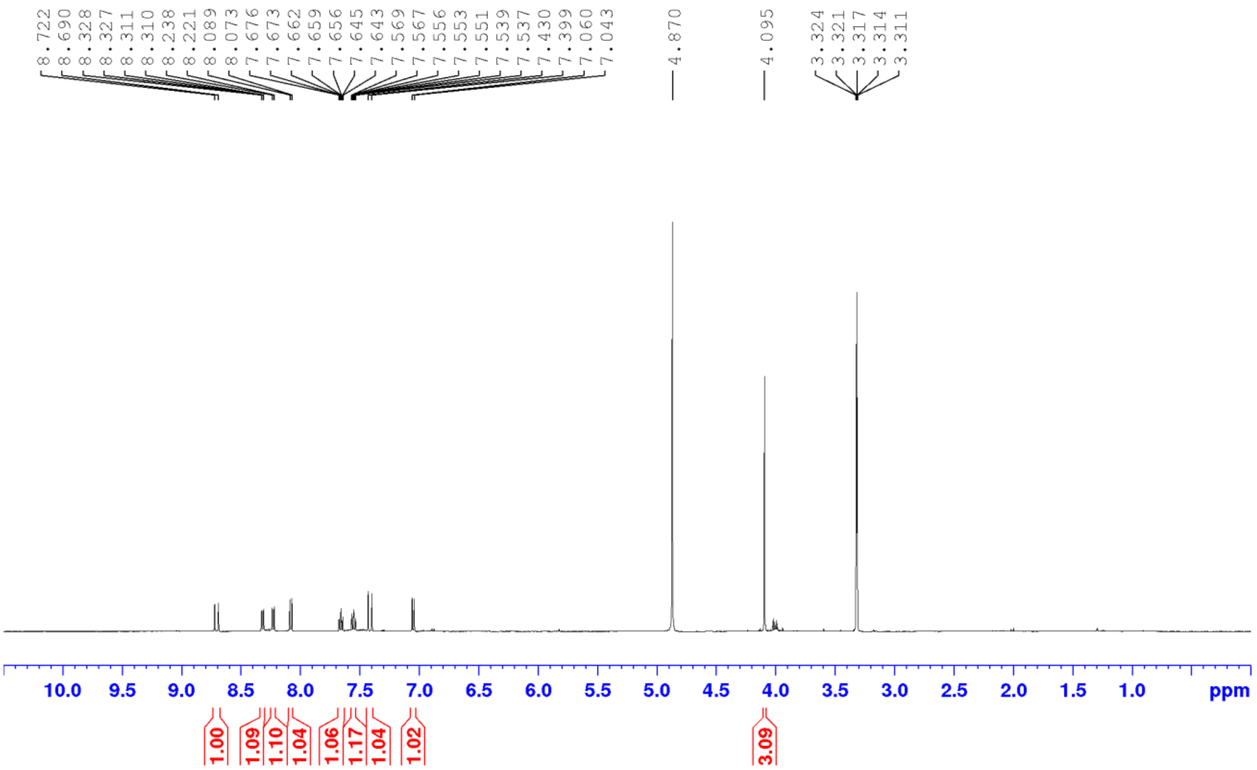

^{1}H NMR spectra of **1d** in CD_3OD

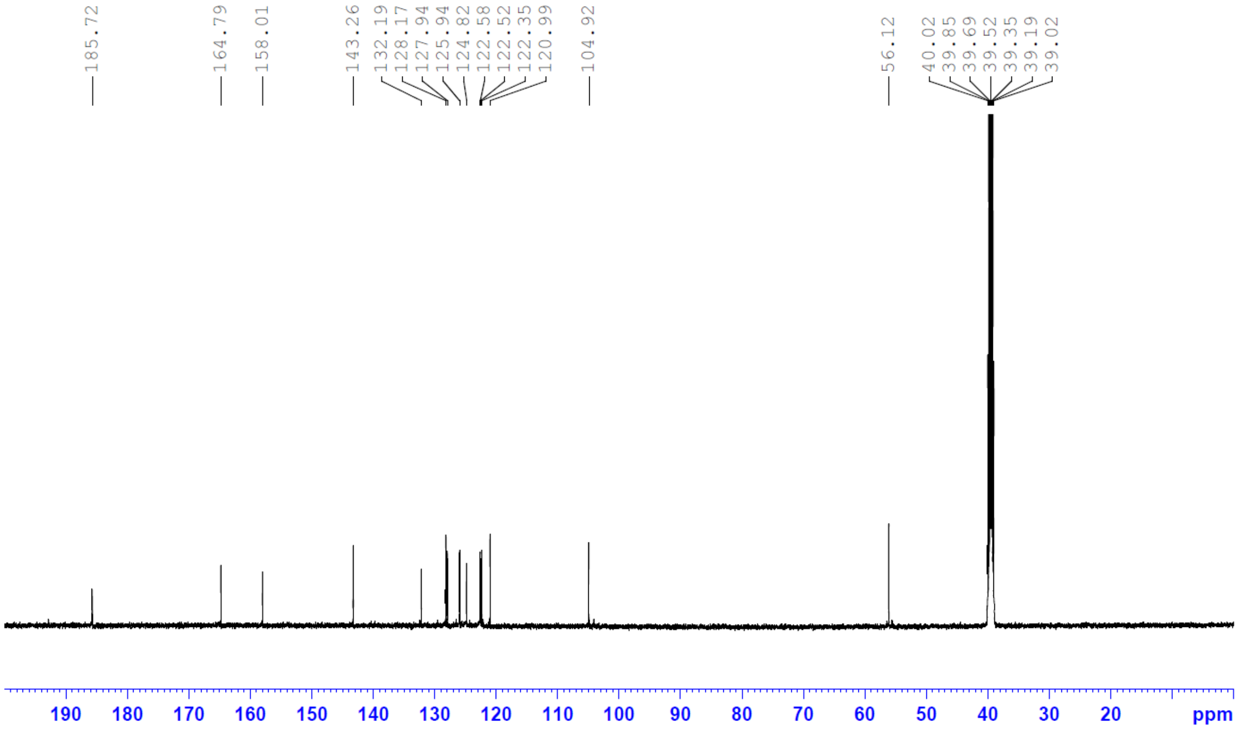

^{13}C NMR spectra of **1d** in CD_3OD

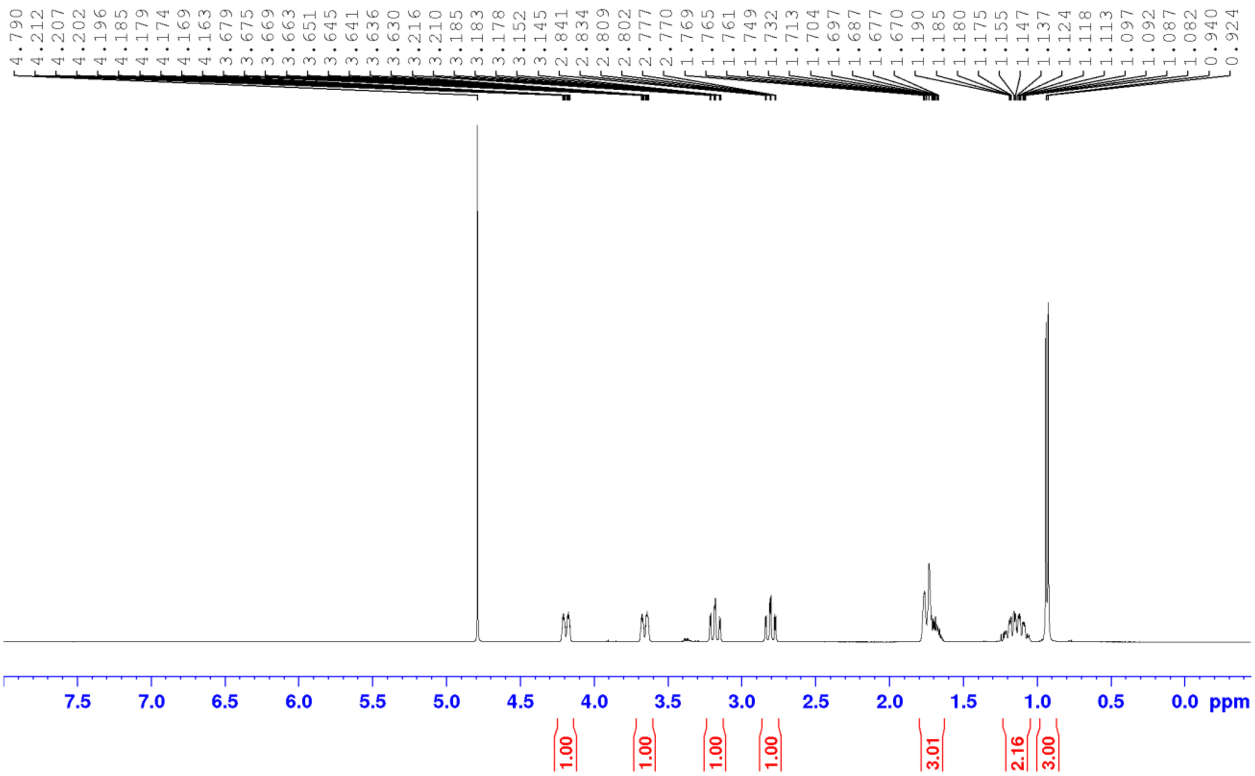

^{1}H NMR spectra of **2a** in D_2O

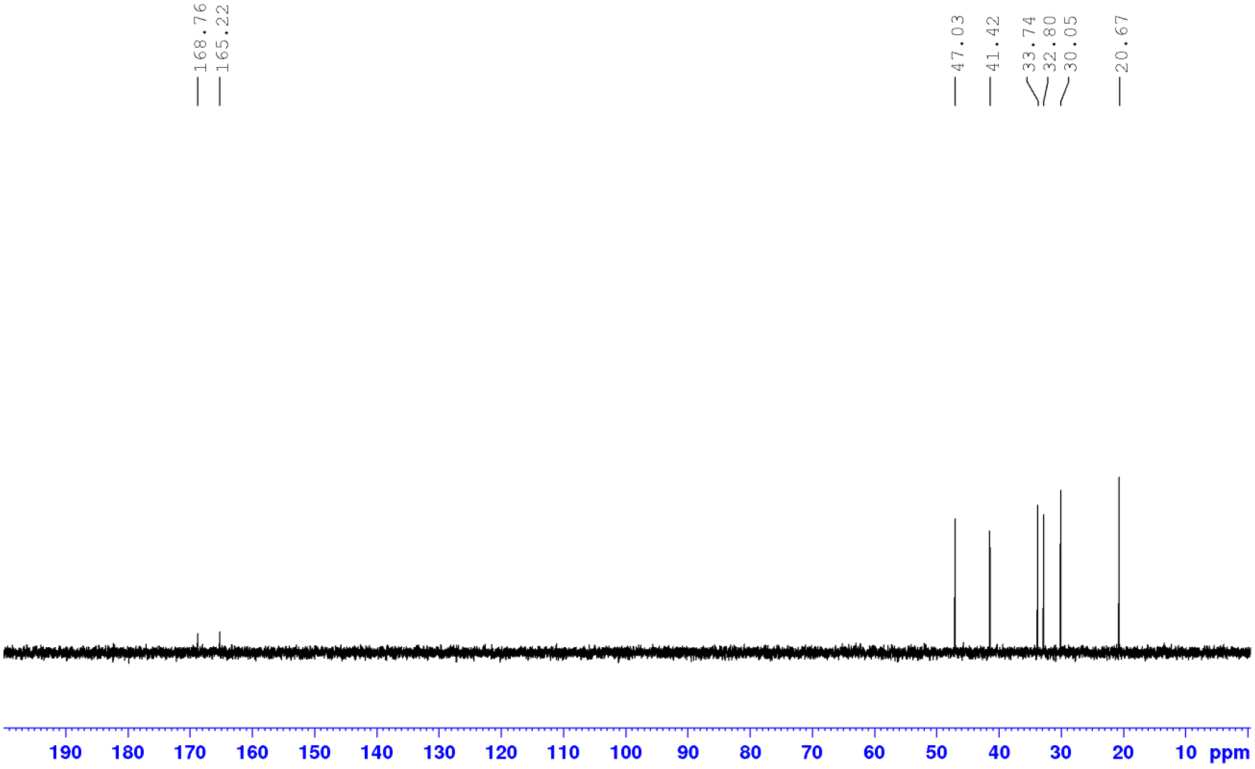

^{13}C NMR spectra of **2a** in D_2O

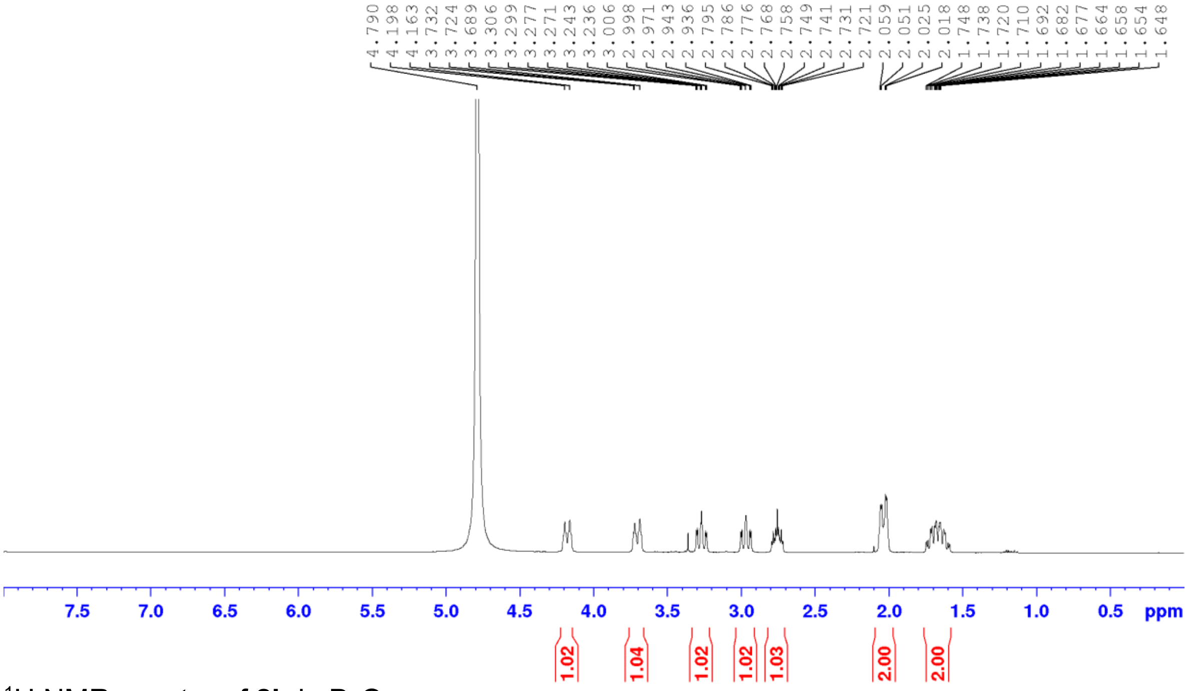

^{1}H NMR spectra of **2b** in D_2O

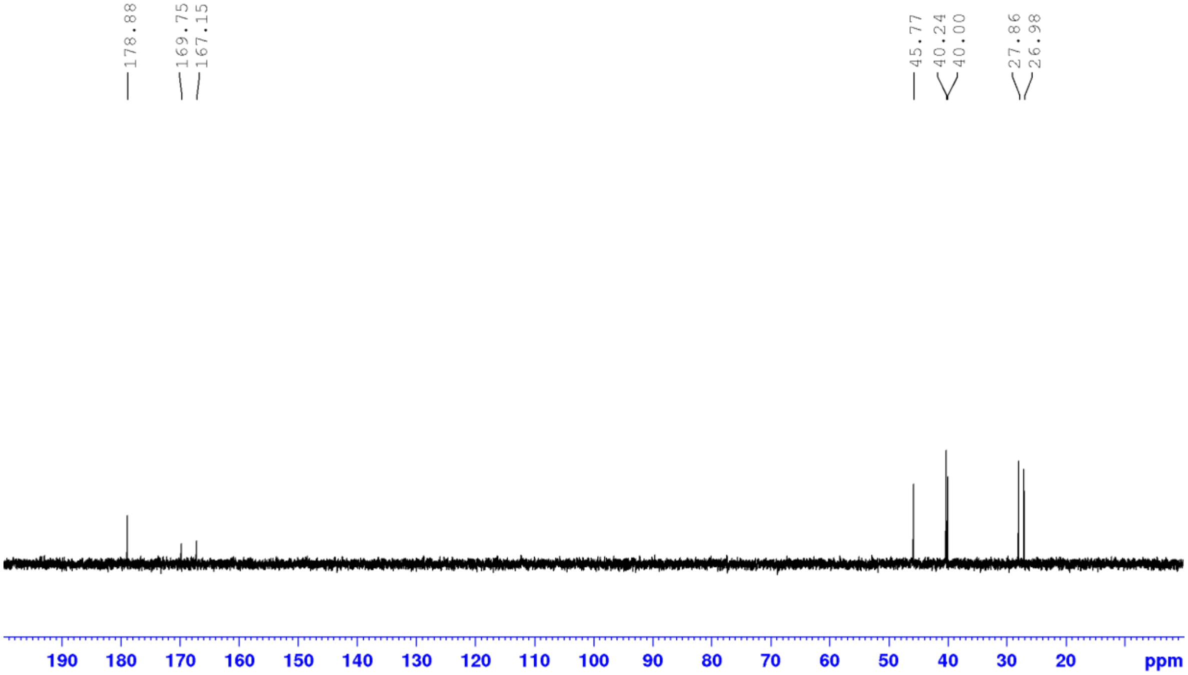

^{13}C NMR spectra of **2b** in D_2O

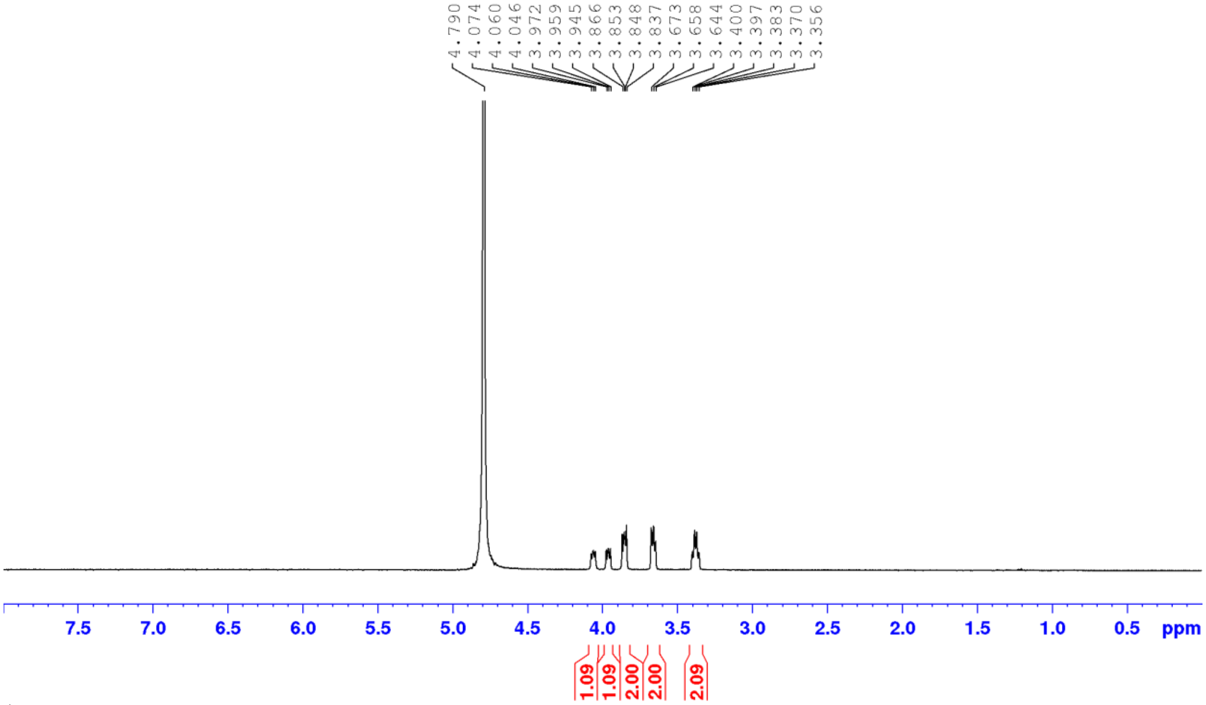

^{1}H NMR spectra of **2c** in D_2O

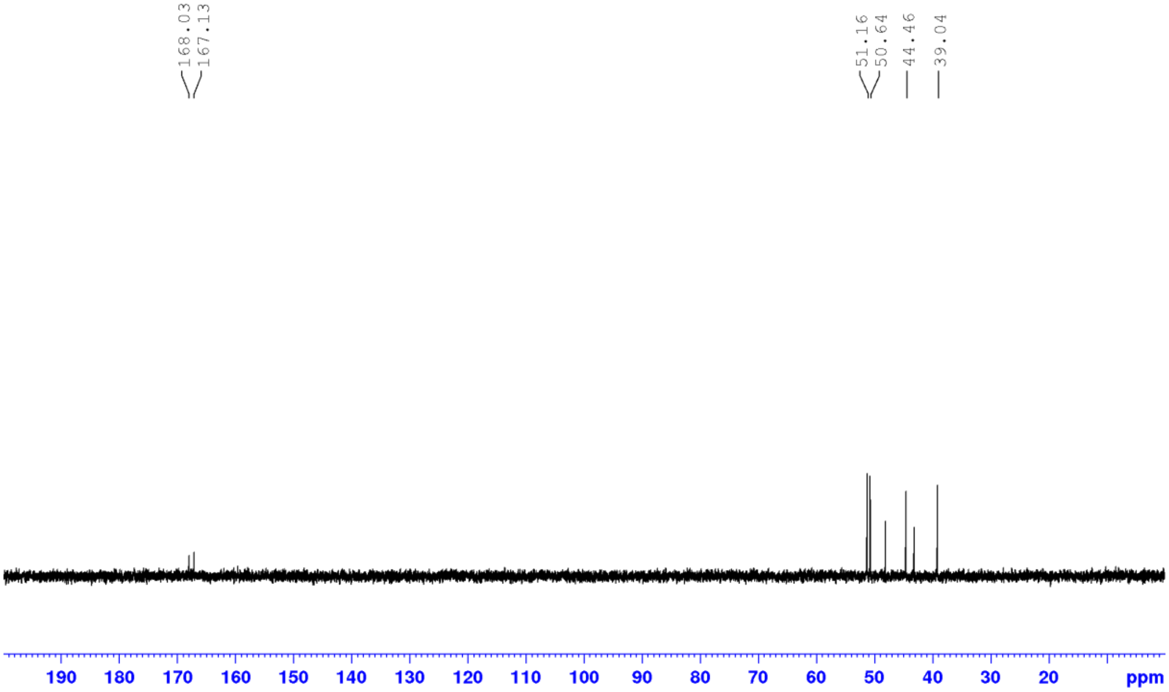

^{13}C NMR spectra of **2c** in D_2O

Supplementary Figures

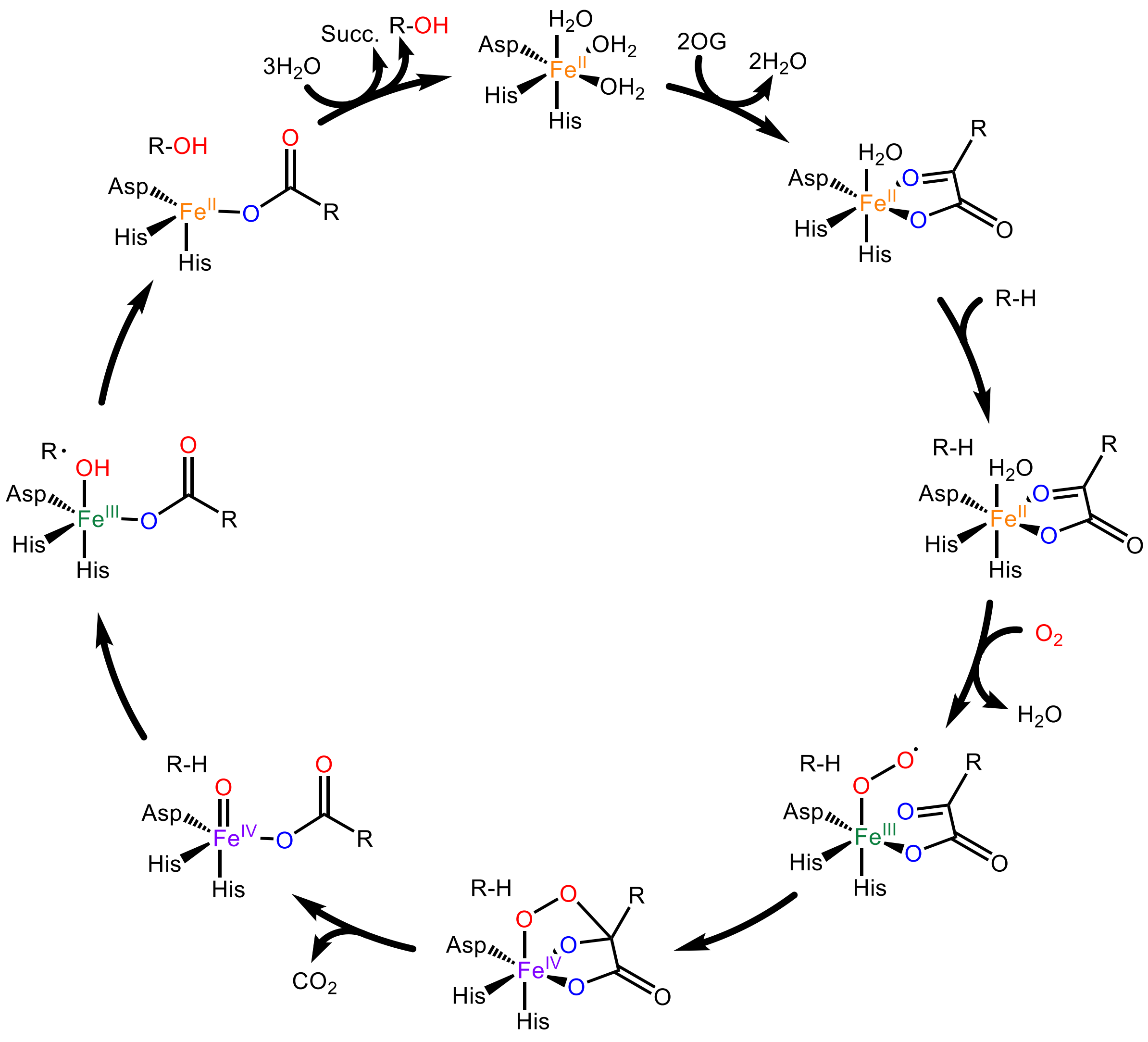

Figure S1. Consensus catalytic cycle of 2OG-dependent NHFe enzymes.

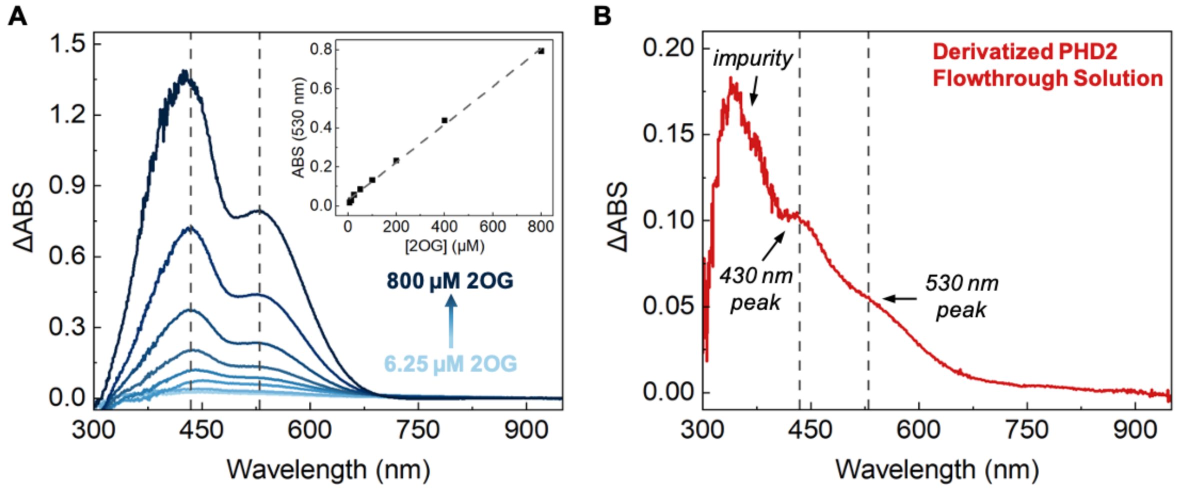

Figure S2. A) UV/Vis spectra for increasing concentrations of 2,4-DNPH derivatized 2OG standards, and the resulting standard curve from absorbance values at 530 nm (inset). Gray dashed reference lines represent the peak maxima for 2,4-DNPH derivatized 2OG (420 and 530 nm). **B)** UV/Vis spectrum for PHD2 flowthrough solution after derivatization with 2,4-DNPH. Gray dashed reference lines represent the peak maxima for 2,4-DNPH derivatized 2OG (420 and 530 nm). Arrows label notable spectral features.

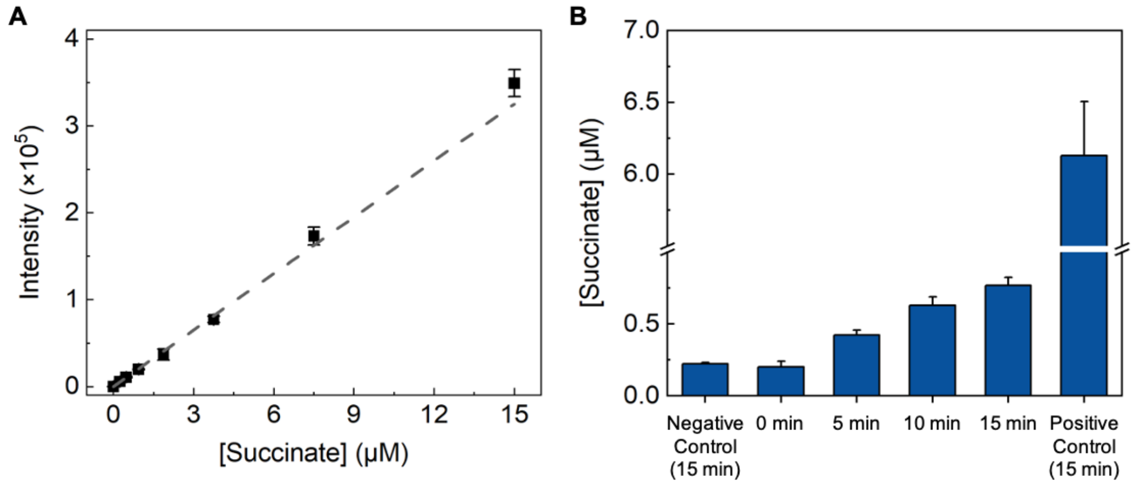

Figure S3. A) Succinate Glo^{TM} assay standard curve generated from succinate standards. **B)** Succinate concentrations from PHD2 reactions determined by Succinate Glo^{TM} assay. Negative control was absent of PHD2 and positive control contained PHD2 and 10 μM 2OG.

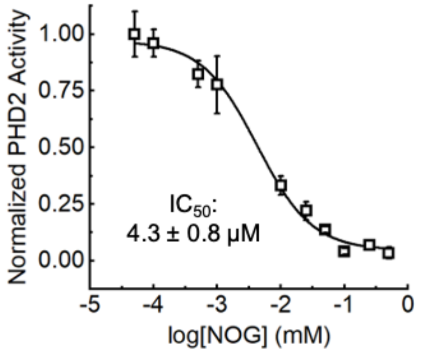

Figure S4. Dose-response curve for NOG and IC_{50} value determined by hydroxylation assay. Error bars represent SD (n = 3)

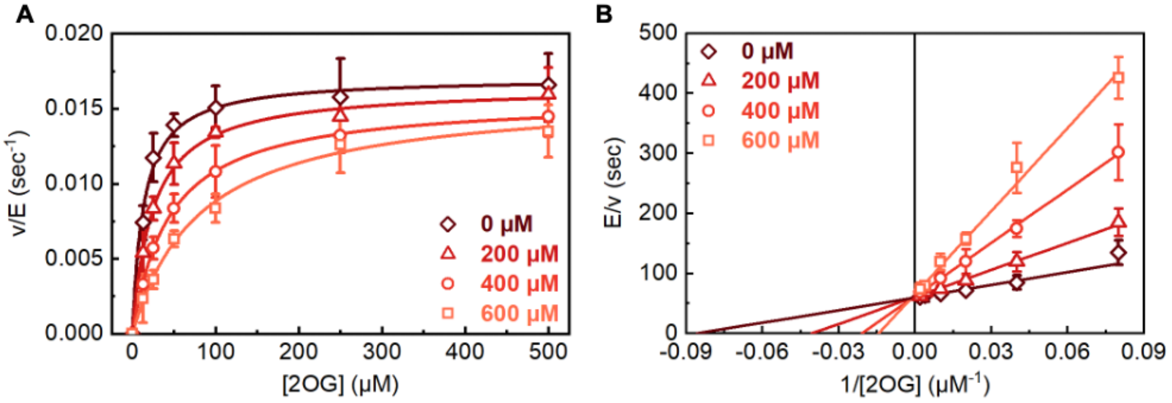

Figure S5. A) 2OG-dependent steady-state kinetics for inhibition of PHD2 at different concentrations of **1a** (0, 200, 400, and 600 μM). Activity was monitored by hydroxylation assay. Error bars represent SD (n = 3). **B)** Lineweaver-Burke plots for inhibition of PHD2 at different concentrations of **1a** (0, 200, 400, and 600 μM). Error bars represent SD (n = 3).

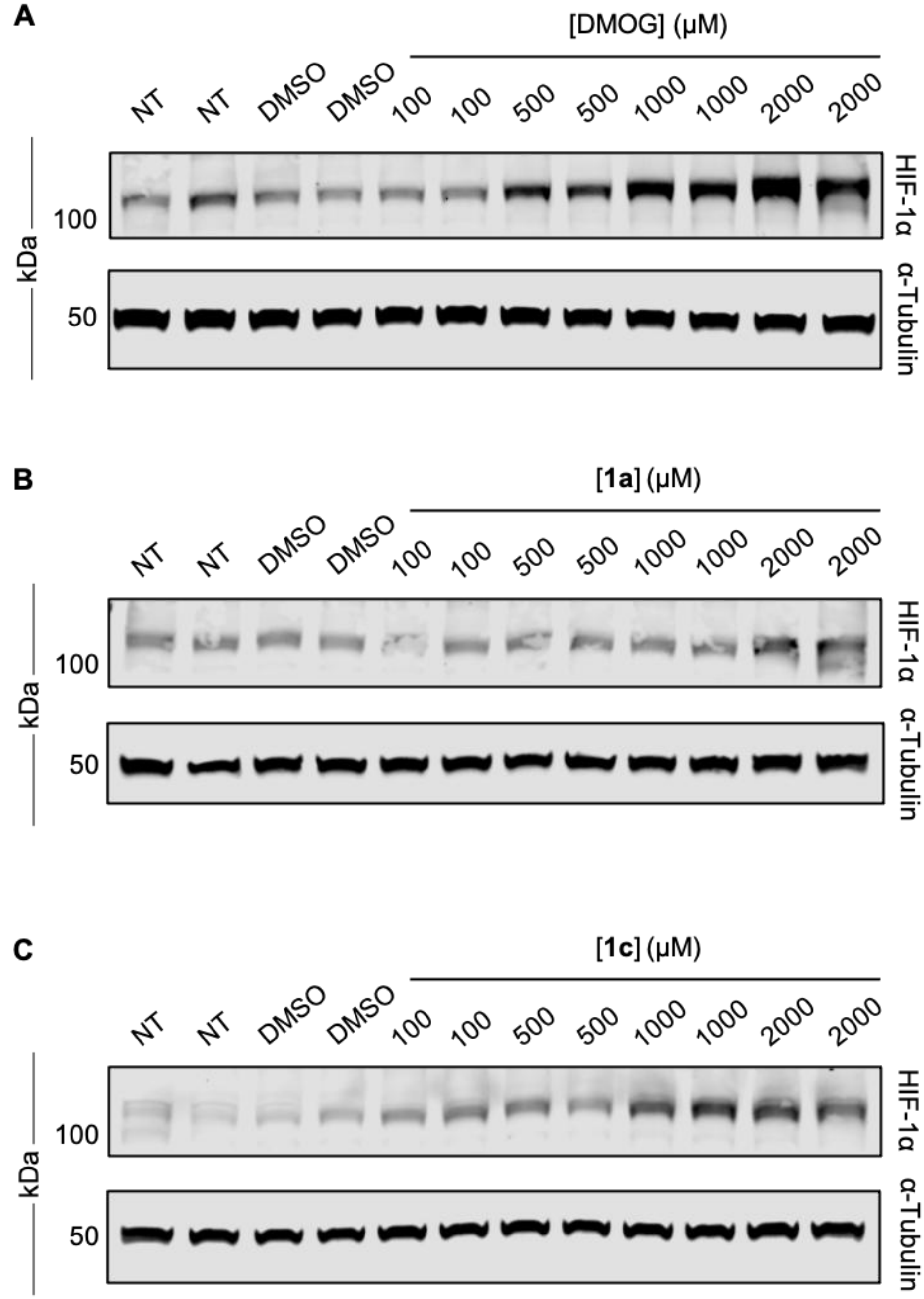

Figure S6. Immunoblot monitoring of HIF-1α levels of HEK-293T cells after treatment with 2OG analogues. α-Tubulin was stained as a loading control. **A)** DMOG dose-response. **B) 1a** dose-response. **C) 1c** dose-response.

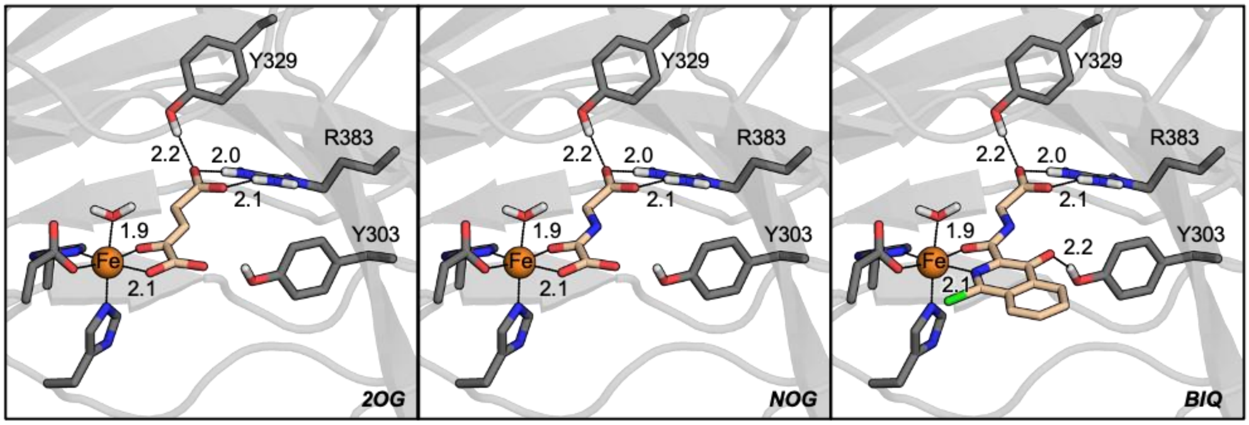

Figure S7. Binding modes of 2OG, NOG, and BIQ predicted from docking calculations. Docked molecules are shown in tan. Dotted lines are contacts that the analogues make with residues in the binding pocket. Labeled distances are in angstroms (Å).

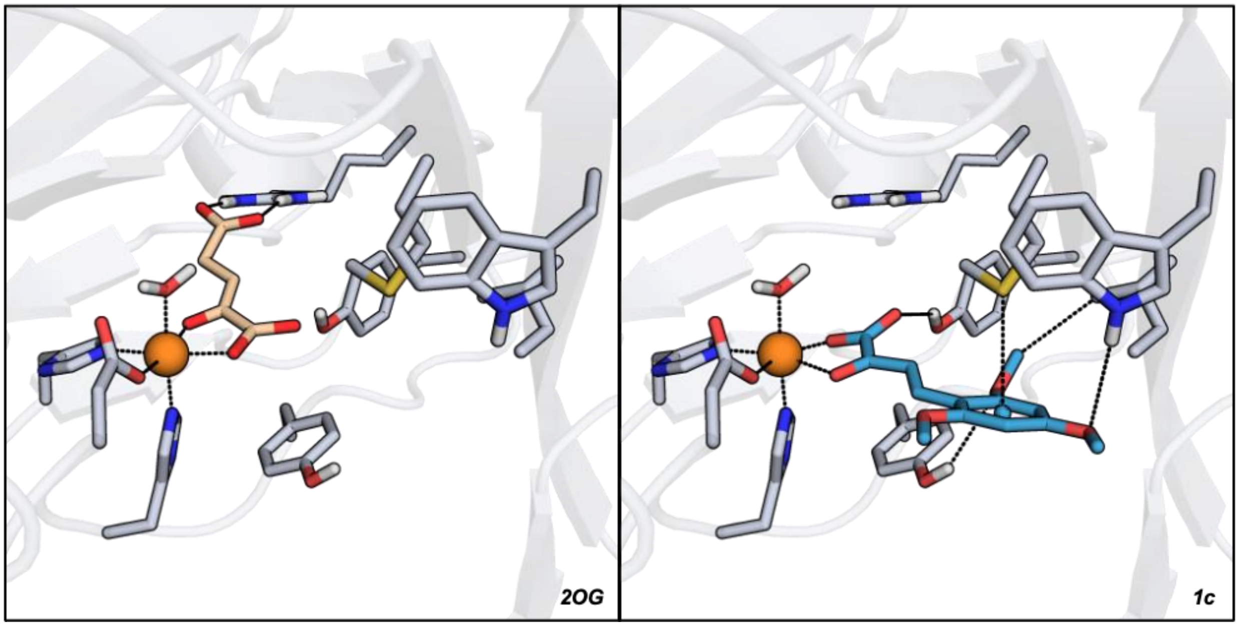

Figure S8. Binding modes of 2OG and **1c** predicted from docking calculations. Comparison of the binding modes indicate that **1c** binds in a "flipped" conformation relative to 2OG. Dotted lines are contacts that the analogues make with residues in the binding pocket.

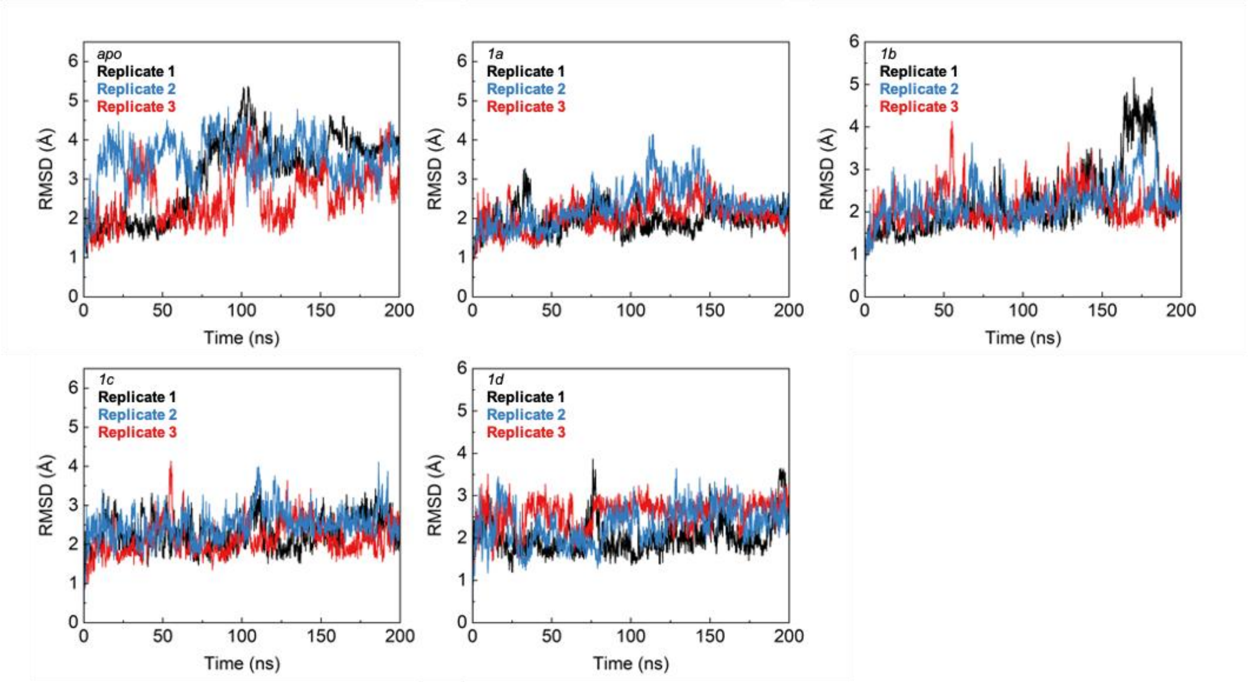

Figure S9. Triplicate RMSD profiles from MD trajectories (200 ns) of apo-PHD2 and PHD2 bound to analogues.

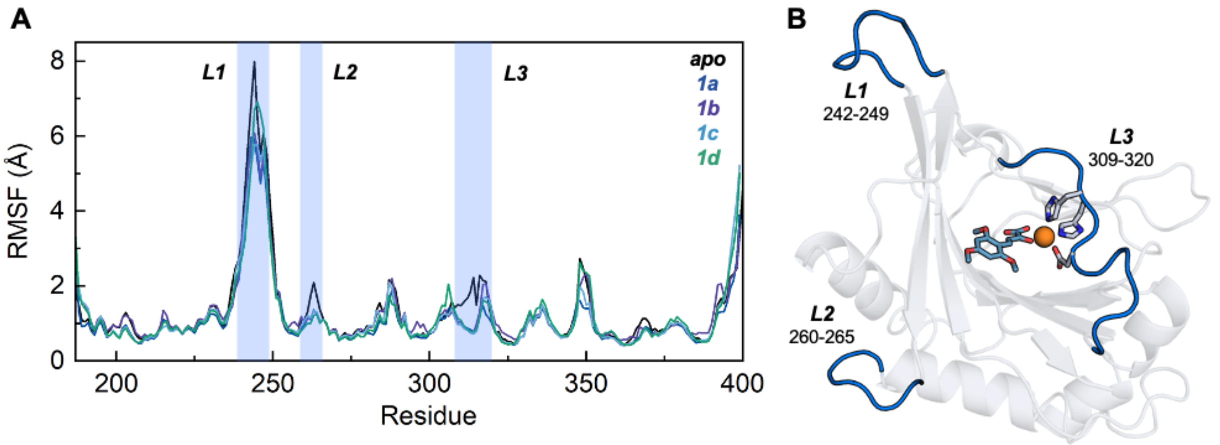

Figure S10. A) Average per-residue RMSF profiles (n = 3) from MD trajectories of apo-PHD2 and PHD2 bound to analogues. Light blue shaded regions represent loops of the protein (L1-3) that differ significantly between the apo and bound structures. **B)** Protein structure of PHD2 bound to **1c**. Opaque blue loops (L1-3) correspond to regions of the protein that differ significantly in average per-residue RMSF between the apo and bound structures.

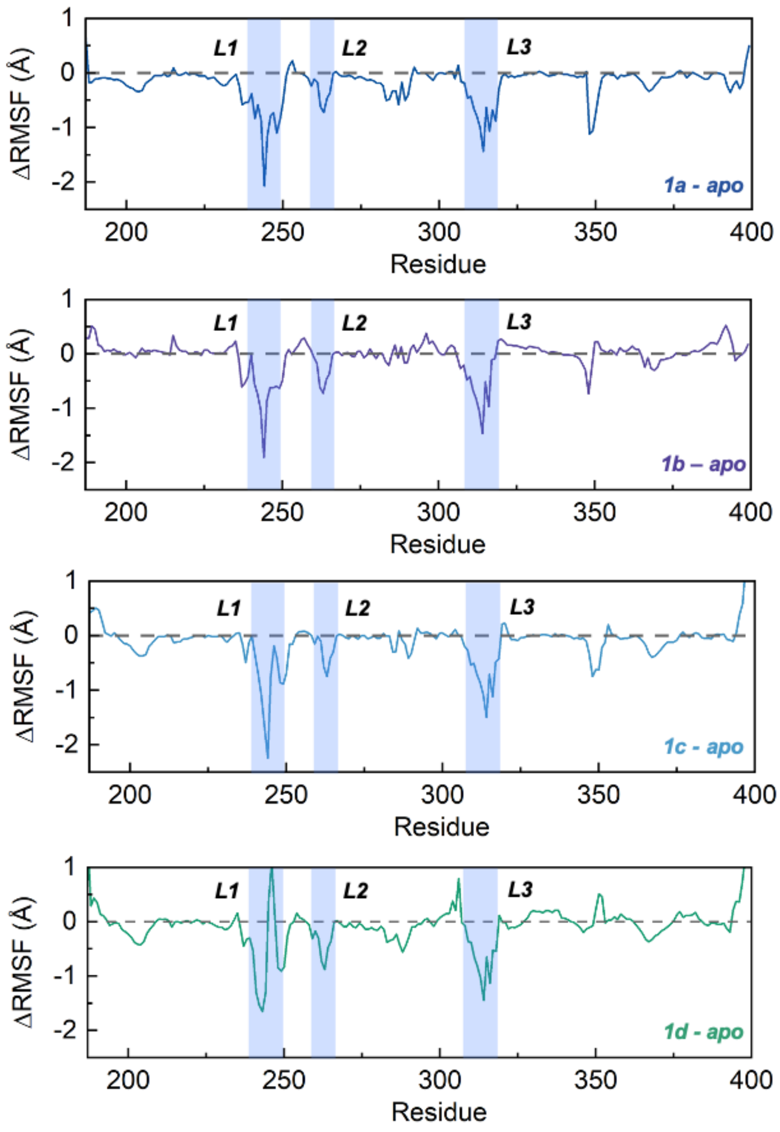

Figure S11. Difference average per-residue RMSF profiles (n = 3) from MD trajectories of apo-PHD2 and PHD2 bound to analogues. Light blue shaded regions represent loops of the protein (L1-3) that differ significantly between the apo and bound structures.

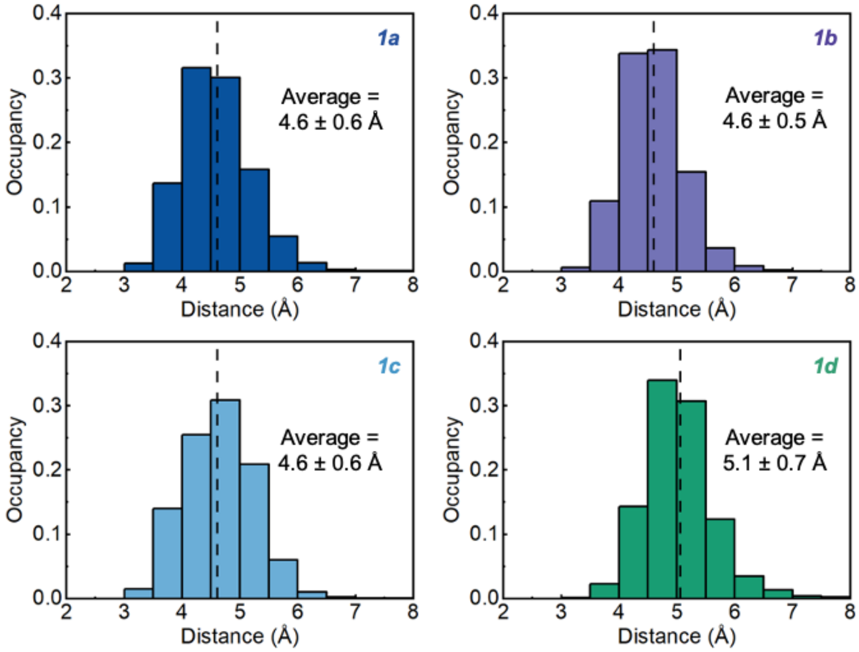

Figure S12. Histograms of distances (Å) between the sulfur of M299 and the aryl group of the analogues sampled from MD trajectories.

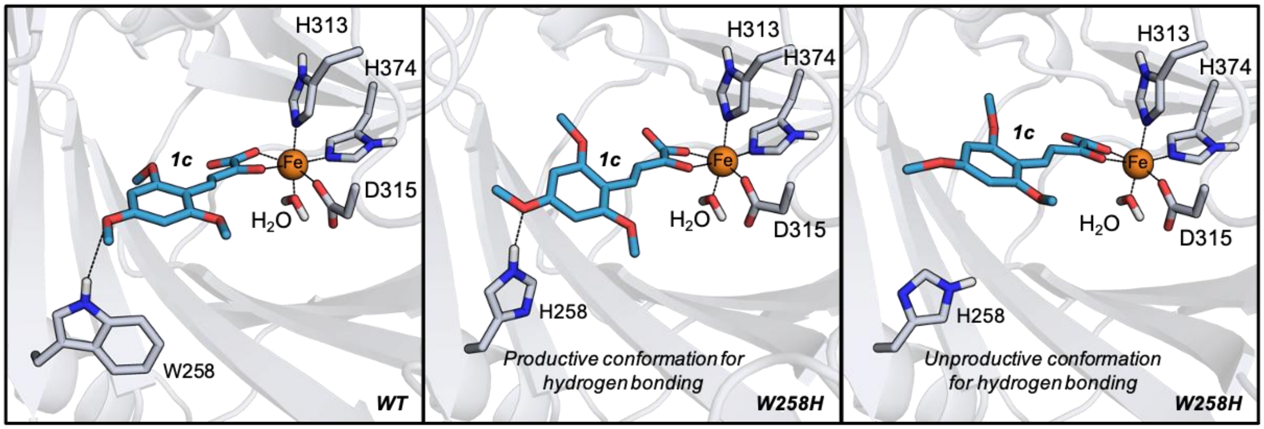

Figure S13. Examples of conformations occupied by W258 (WT PHD2) and H258 (W258H PHD2) from MD simulations with analogue **1c**. The side chain of H258 rotates 180° around the $C_\beta - C_\gamma$ bond which positions it in an unproductive conformation to hydrogen bond to the *p*-methoxy group of 1c. W258 does not rotate in this manner.

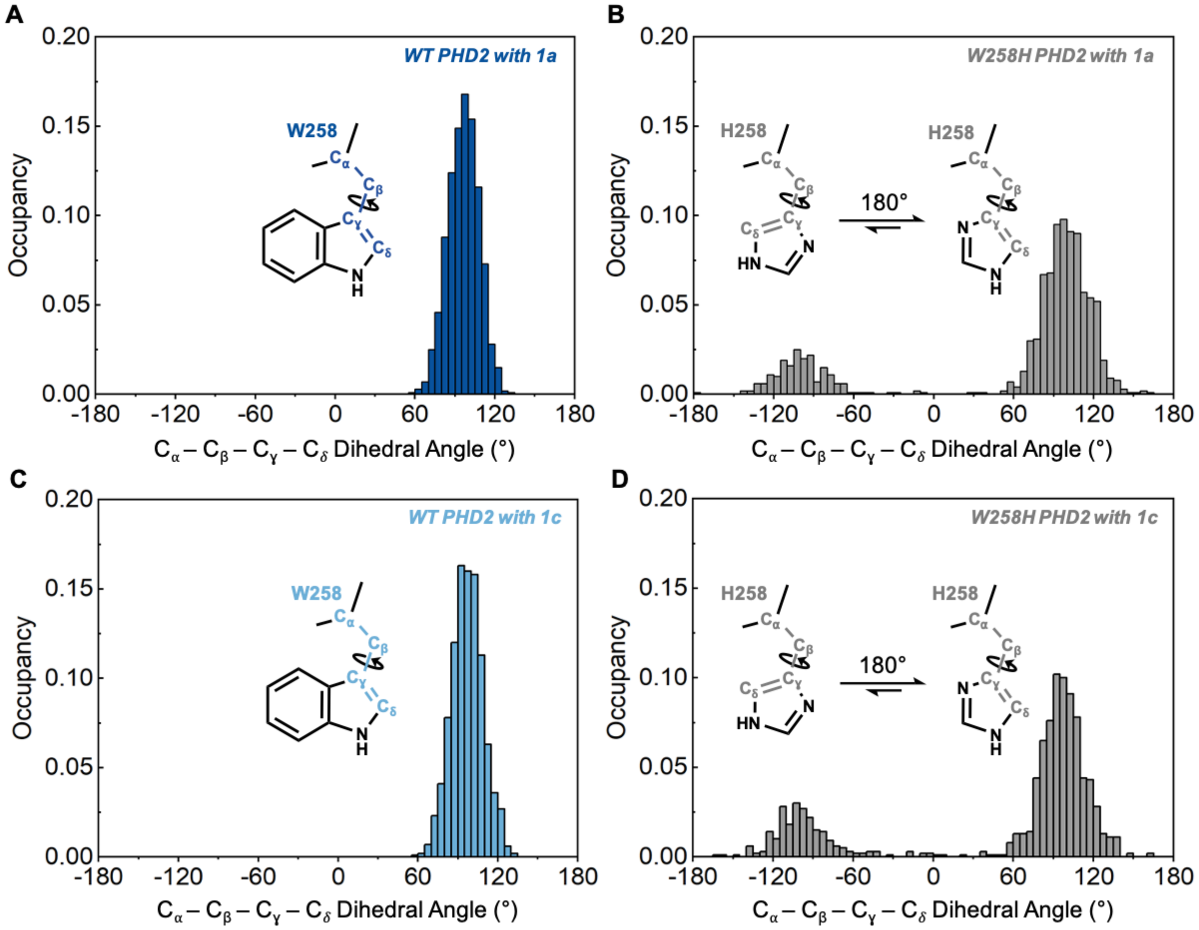

Figure S14. Histograms of the C_α – C_β – C_γ – C_δ dihedral angle for residue 258 across MD simulations. Atoms within the structures of tryptophan and histidine are colored to highlight which dihedral bond is being sampled. **A)** W258 from WT PHD2 bound to **1a**. **B)** H258 from W258H PHD2 bound to **1a**. **C)** W258 from WT PHD2 bound to **1c**. **D)** H258 from W258H PHD2 bound to **1c**.

Supplementary Tables

Table S1. 2OG-dependent kinetic constants (± SD) of PHD2 after treatment with **1a**.

[1a] (µM)	K_M(2OG) (µM)	k_{cat} (s^{-1})
0	13 ± 2	0.0171 ± 0.0003
200	24 ± 2	0.0164 ± 0.0002
400	45 ± 1	0.0157 ± 0.0009
600	81 ± 8	0.0160 ± 0.0005

Table S2. 2OG-dependent kinetic constants (± SD) of PHD2 after treatment with **1c**.

[1c] (µM)	K_M(2OG) (µM)	k_{cat} (s^{-1})
0	13 ± 2	0.0171 ± 0.0003
100	27 ± 5	0.0161 ± 0.0007
200	47 ± 5	0.0161 ± 0.0009
300	80 ± 15	0.016 ± 0.001

Table S3. Primers used in site-directed mutagenesis

Mutation	Forward (5' → 3')	Reverse (5' → 3')
I256A	GAGGCGATAAGGCCACCTGGATCGA	GGATGTCCTTGGACGAGTCACTCTTC
W258F	GATAAGATCACCTTCATCGAGGGCAAG	GCCTCGGATGTCCTTGGACGAGT
W258H	GATAAGATCACCCACATTGAGGGCAAGG	ACCGCGGATGTCTTTGCTCGAGT
M299A	CGGACGAAAGCCGCGGTTGCTTGTTAT	GCCATTGATTTTGTAGCTGCCCAGCT
Y310T	GAACGGGTACTGTACGTCATGTTG	GACGTACAGTACCCGTTCCATTGCC

References

[56] R. Chowdhury, I. K. H. Leung, Y. M. Tian, M. I. Abboud, W. Ge, C. Domene, F. X. Cantrelle, I. Landrieu, A. P. Hardy, C. W. Pugh, P. J. Ratcliffe, T. D. W. Claridge, C. J. Schofield, *Nat Commun* **2016**, *7*, 12673.

[57] M. A. Mingroni, V. Chaplin Momaney, A. N. Barlow, I. Jaen Maisonet, M. J. Knapp, in *Methods in Enzymology*, Elsevier, **2023**, pp. 363–380.

[58] R. A. Friesner, J. L. Banks, R. B. Murphy, T. A. Halgren, J. J. Klicic, D. T. Mainz, M. P. Repasky, E. H. Knoll, M. Shelley, J. K. Perry, D. E. Shaw, P. Francis, P. S. Shenkin, *J. Med. Chem.* **2004**, *47*, 1739–1749.

[59] P. K. Windsor, S. P. Plassmeyer, D. S. Mattock, J. C. Bradfield, E. Y. Choi, B. R. Miller, B. H. Han, *IJMS* **2021**, *22*, 2888
